# How Far Are We from Prescribing Fasting as Anticancer Medicine?

**DOI:** 10.3390/ijms21239175

**Published:** 2020-12-01

**Authors:** Maria V. Deligiorgi, Charis Liapi, Dimitrios T. Trafalis

**Affiliations:** Department of Pharmacology—Clinical Pharmacology Unit, Faculty of Medicine, National and Kapodistrian University of Athens, 115 27 Goudi, Athens, Greece; cliapi@med.uoa.gr (C.L.); dtrafal@med.uoa.gr (D.T.T.)

**Keywords:** fasting, fasting mimicking diet, short-term fasting, starvation chemotherapy efficacy, chemotherapy toxicity, differential stress resistance, differential stress sensitization

## Abstract

(1) Background: the present review provides a comprehensive and up-to date overview of the potential exploitation of fasting as an anticancer strategy. The rationale for this concept is that fasting elicits a differential stress response in the setting of unfavorable conditions, empowering the survival of normal cells, while killing cancer cells. (2) Methods: the present narrative review presents the basic aspects of the hormonal, molecular, and cellular response to fasting, focusing on the interrelationship of fasting with oxidative stress. It also presents nonclinical and clinical evidence concerning the implementation of fasting as adjuvant to chemotherapy, highlighting current challenges and future perspectives. (3) Results: there is ample nonclinical evidence indicating that fasting can mitigate the toxicity of chemotherapy and/or increase the efficacy of chemotherapy. The relevant clinical research is encouraging, albeit still in its infancy. The path forward for implementing fasting in oncology is a personalized approach, entailing counteraction of current challenges, including: (i) patient selection; (ii) fasting patterns; (iii) timeline of fasting and refeeding; (iv) validation of biomarkers for assessment of fasting; and (v) establishment of protocols for patients’ monitoring. (4) Conclusion: prescribing fasting as anticancer medicine may not be far away if large randomized clinical trials consolidate its safety and efficacy.

## 1. Introduction

Since time immemorial, voluntary fasting has been a part of religious rituals integrated in various ethno-cultural contexts, including Christianity, Hinduism, Judaism, Buddhism, Islam, and African animistic religions, because of its biological and spiritual threads. In 1909, Moreschi reported for the first time the abrogation of growth of transplanted tumors in underfed mice [1], paving the way for McCay et al. to connect the underfeeding with longevity in rats, almost thirty years later [2]. Pursuing fasting as a “fountain of youth” was initially scrutinized by the forerunners of aging research, while being applauded after the 1980s [3,4]. In 2002, the first guidelines were released concerning fasting as a therapeutic procedure in specialized fasting departments of hospitals in the context of integrative medicine. Due to sparsity of data, cancer was not included in the empirically proven indications for fasting in the updated version of these guidelines in 2013 [5]. Since then, implementing fasting in oncology is a rapidly evolving field of research [6,7].

With 1,806,590 estimated new cases and 606,520 estimated deaths worldwide in 2020, establishment of affordable and feasible interventions to decrease the cancer burden is a priority [8]. Although the scientific breakthrough of the last decade yielded cutting-edge anticancer therapies [9], chemotherapy will remain a tangible treatment option for the coming decades. However, two major hurdles to the optimal response rates to chemotherapy are the treatment-associated toxicity and the chemoresistance. To overcome these barriers, the development of novel strategies could capitalize on the distinct bioenergetics of cancer, a notion conceived in the first half of the last century and integrated in the pathogenesis of cancer in the postgenome era [10]. Initially considered as a reflection of oncogenic reprogramming, the metabolic reprogramming plays per se a fundamental role in genetic and epigenetic events [11,12,13] and chemoresistance [14].

**Figure 1:**
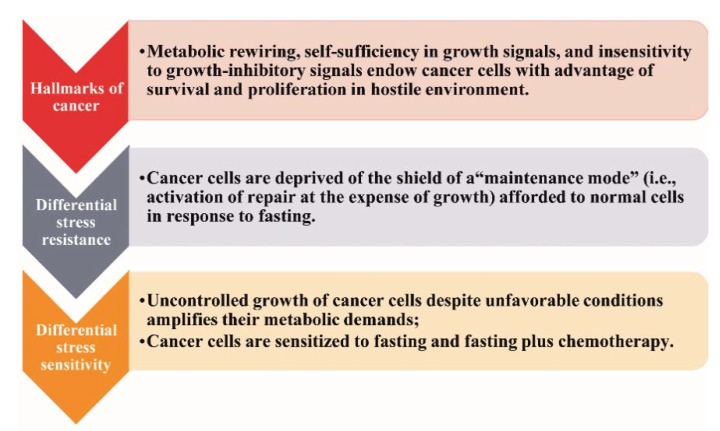
The differential stress response.

So far, the only widely accepted nutritional intervention strategies implemented in cancer patients address the cancer-related malnutrition and the counteraction of obesity [15,16,17]. Currently, the potential anticancer efficacy of fasting has gained wide clinical and scientific interest [18]. The rationale is the differential stress response, as illustrated in Figure 1 [6].

In response to restriction of nutrients, normal cells enter a “maintenance mode” characterized by activation of catabolic processes and mechanisms of repair to preserve the integrity of the genome and the proteome at the expense of proliferation and growth [6,7]. Cancer cells are deprived of this “shield” (“differential stress resistance”), thereby being vulnerable to fasting or combining fasting with chemotherapy (“differential stress sensitization”) [6,7].

Among innumerous dietary interventions of food deprivation, three are increasingly correlated with a beneficial effect on metabolism and a potential anticancer activity, namely the fasting, the fasting mimicking diet (FMD), and the calorie restriction (CR) [19,20,21,22,23,24,25,26,27,28,29,30,31,32,33,34,35,36,37,38,39,40,41,42,43]. Table 1 depicts a glossary of terms of the most applied interventions of food deprivation.

The present narrative review provides a comprehensive overview of evidence, challenges, and future perspectives concerning the exploitation of food deprivation as an anticancer strategy. This concept is principally rationalized by mounting nonclinical and clinical evidence sustaining that fasting can increase the tolerability and the efficacy of chemotherapy.

## 2. The Response to Fasting

So far, identification of a single signaling cascade, molecular mechanism, or genetic event responsible for the response to fasting and thus the effects of fasting in the setting of oncology seems daunting. Instead, a more practical approach draws on an interplay among hormonal, metabolic, and cellular alterations assigned to counteract the metabolic stress. Indeed, the response to fasting is a finely tuned network integrating hormones-driven metabolic adaptations, which modulate key molecular cascades, dictate cellular alterations, and result in acquisition of stress resistance phenotypes.

**Figure 2:**
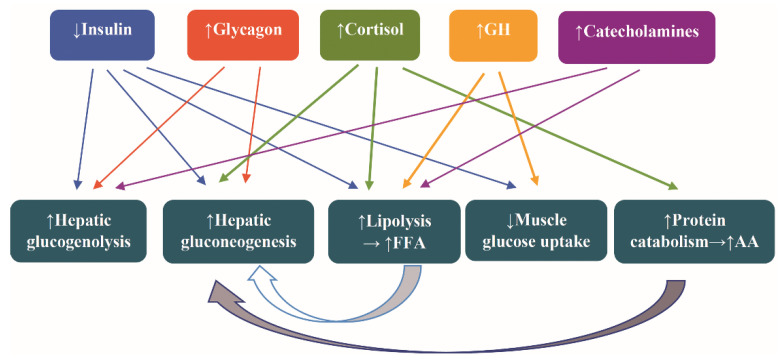
Hormonal and metabolic alterations as a counterregulatory response to fasting. Abbreviations: AA, amino acids; FFA, free fatty acids; and GH, growth hormone. Arrow ↑: increase; arrow ↓: decrease.

The hormonal alterations assigned to counteract the fasting are increased secretion of catecholamines, glucagon, cortisol, and growth hormone (GH) and decreased secretion of insulin. The attendant metabolic alteration are increased glycogenolysis, lipolysis, hepatic gluconeogenesis, and protein catabolism and decreased muscle uptake of glucose [44] (Figure 2).

Notably, the levels of insulin-like growth factor 1 (IGF-1) decline, and the biological activity of IGF-1 is further compromised due to increased levels of insulin-like growth factor binding protein 1 (IGFBP1). Finally, increased levels of adiponectin stimulate the fatty acid breakdown.

Increased hepatic glycogenolysis and hepatic gluconeogenesis are the first and second, respectively, steps to counteract hypoglycemia. After depletion of stored glycogen, which occurs usually 24 h after initiation of fasting, the fatty acids serve as the main fuels for most tissues, while the brain relies on glucose gained through gluconeogenesis—approximately 80 g/day—using ketone bodies, fat-derived glycerol, and amino acids (AA) [44]. The brain utilizes also the ketone bodies β-hydroxybutyrate and acetoacetate [45]. After a week or more of starvation, the fat-derived β-hydroxybutyrate is the prevailing ketone, whereas glucose production is lowered [46].

The neuroendocrine component of the response to fasting comprises the hypothalamic arcuate neurons that synthesize the neuropeptide Y (NPY) and the agouti related peptide (AGRP). These neuropeptides control food intake through sensing alterations in blood glucose, integrating peripheral signals that increase (e.g., ghrelin) or decrease (e.g., insulin, leptin) the systemic response to a metabolic stress [47]. Interestingly, the AgRP and the NPY are expressed not only in the hypothalamic neurons but also in the adrenal chromaffin cells, mediating the crosstalk between the periphery and the hypothalamus in response to fasting [47,48].

The signaling cascades downstream of the fasting-induced reduction of glucose and/or IGF-1 levels are the key mediators of the cellular effects of fasting. Such cascades involve the Ras/Adenylate cyclase (AC)/Protein kinase A (PKA), the mammalian target of rapamycin (mTOR)/S6 kinase 1 (S6K1), and the phosphoinositide 3-kinase (PI3K)/Protein kinase B (Akt)/S6K1. Subsequently, pivotal transcription factors, such as NF-E2-related factor 2 (Nrf2), GIS1, MSN2/4, DAF-16, (Forkhead box protein (FOXO), and Hypoxia-inducible factor 1 (HIF1), are modulated, regulating the expression of proteins involved in adaptive cellular processes, including inhibition of cell proliferation, induction of apoptosis, autophagy, DNA repair, genomic stability, and carcinogen-detoxification [18].

Additional mechanisms potentially orchestrating an antitumor effect of food deprivation have been described in vitro and in vivo, including: (i) increase of adiponectin levels decrease of leptin levels, resulting in significantly higher adiponectin to leptin ratio compared to ad libitum status [49]; (ii) promotion of antitumor immune response [50]; (iii) upregulation of E-cadherin expression via activation of c-Src kinase [51]; (iv) decrease of cytokines, chemokines, metalloproteinases, growth factors [52]; (v) amelioration of insulin sensitivity [53]; (vi) increase of level of activated caspase-3 [54]; and (vii) phosphorylation of H2AX (a variant of the histone H2A family), a step critical not only for repair of damaged DNA but also for initiation of apoptosis [54].

Although initially applauded, the fasting-induced activation of apoptosis should be interpreted cautiously in view of the intriguing clinical outcome. In fact, nutrient deprivation exerts a proapoptotic effect through BH3-only protein Puma, but cancer cells may resist to this effect via the post-translational stabilization of p21, which prevents Puma and its downstream effector Bax from triggering the mitochondrial apoptotic pathway [55].

Likewise, the clinical outcome of the fasting-induced induction of autophagy is elusive. Autophagy is a lysosome-mediated process, ensuring cellular integrity and maintenance of energy balance, eliminating dysfunctional organelles and proteins in response to DNA damage, but it plays an equivocal role in the cell fate. While protecting normal cells from oxidative stress and DNA damage, autophagy enables the survival and growth of cancer cells in adverse conditions, such as hypoxia and nutrient starvation; however, under certain conditions, it induces programmed cancer cell death [56,57]. Indeed, Poly (ADP-ribose) polymerase 1 (PARP-1), the factor that stimulates the starvation-induced autophagy in response to oxidative stress and DNA damage, activates two distinct pathways: The activation of PARP-1 is responsible for the prosurvival impact of autophagy on cancer cells mediated via the liver kinase B1 (LKB1)/AMP activated protein kinase (AMPK)/mTOR pathway, but the overactivation of PARP-1 promotes ATP depletion and necrotic cell death [58].

## 3. The Interrelationship of Fasting with Oxidative Stress: Exemplification of Hormesis

Although fasting intervenes in the redox balance, the clinical relevance is elusive mainly due to the two-faced profile of the reactive oxygen species (ROS), the key player of oxidative stress. ROS are chemically reactive species containing oxygen. Superoxide—the precursor of most other reactive oxygen species—is formed by the univalent reduction of triplet-state molecular oxygen (^3^O_2_) enzymatically (e.g., by NAD(P)H oxidases) or nonenzymatically. ROS are generated from endogenous sources (e.g., mitochondria, peroxisomes, lipoxygenases, NADPH oxidases, cytochrome P450), antioxidant defenses (e.g., enzymatic and nonenzymatic systems), and exogenous sources (ultraviolet light, ionizing radiation, chemotherapy, and environmental toxins) [59,60]. Recognized less than 50 years ago, ROS were initially incriminated for subverting the genome stability and the cellular integrity, contributing to aging and numerous diseases, including cancer. Increased intracellular ROS levels foster acquisition of the hallmarks of cancer and increased extracellular ROS levels favor the multifocality and the metastatic potential of tumors [59,61]. However, low/moderate levels of ROS are crucial for physiological functions, including modulation of vascular tone, sensing of oxygen tension, regulation of oxygen concentration, signal transduction from membrane receptors (e.g., the antigen receptor of lymphocytes), and responses to oxidative stress, resulting in maintenance of redox homeostasis [59,62].

In the 21st century, the conventional concept that the beneficial effects of fasting are ascribed to reduced ROS due to blunted metabolism is challenged, given that the latter requires a long time to accrue. Instead, it is postulated that the beneficial effects of fasting are ascribed to rapid metabolic and immunological response, triggered by a temporary increase in oxidative free radical production [62,63]. In fact, there is an optimal level of cellular ROS production, which yields a beneficial effect on the health span [64]. This phenomenon exemplifies the notion of hormesis. The term “hormesis”—derived from the Greek word “hormo” (excite)—was introduced in biology in 1943 by C. Southam and J. Ehrlich to describe the fact that extracts from the Red Cedar tree stimulate the fungal growth at low doses, while attenuating growth at higher doses. Indeed, lethal stressors applied in doses lower than those having a detrimental effect elicit an adaptive stress-resistant status of an organism (or a cell), empowering survival in case of exposure to devastating doses [65]. Accordingly, deliberate exposure of biological systems to the mild stress of nutrient deprivation stimulates the systems of maintenance and repair, yielding beneficial hormetic effects [66,67,68].

Within this framework, nutrient deprivation elicits a dynamic process governed by activation of energy-sensing signaling transduction cascades assigned to dictate the rewiring of metabolic pathways. Core components of this response are: (i) the AMPK acting as an intracellular energy sensor [69,70]; (ii) the mammalian target of rapamycin (mTOR), which initiates the translation phosphorylating two regulatory proteins, the p70 ribosomal S6 protein kinase (p70^S6K^) and the eukaryotic initiation factor 4E-binding protein-1 (4E-BP1) [71]; and (iii) the general control nondepressible 2 kinase (GCN2), which in response to AA deprivation inhibits translation via phosphorylation of eukaryotic initiation factor (eIF)2α [72,73,74].

Glucose deprivation leads to ATP depletion, resulting in ROS accumulation [75]. Detailed presentation of ROS-induced signaling transduction is a complex issue beyond the scope of the present review. Briefly, ROS induce pivotal signaling cascades, the downstream effectors of which comprise transcription factors, kinases, and translational regulators, which control the expression of target genes implicated in cell proliferation and survival. Such cascades are: (i) the MAPK extracellular signal-regulated kinases (ERK), p38 and Janus kinase (JNK), activating the ETS Like-1 protein (ELK1), the activating transcription factor 2 (ATF2), and the signal transducer and activator of transcription 3 (STAT3); (ii) the PI3K/Akt, inhibiting FOXO, Bad, and glucogen synthase kinase 3 (GSK3), and activating the mTOR complex 1 (mTORC1); and (iii) the Src/Phospholipase D1 (PLD1)/polycystic kidney disease 1 (PKD1)/nuclear factor kappa B (NF-κB) pathway [76,77].

Additionally, ROS activate AMPK, which mediates a metabolic reprogramming credited with reinforcement of catabolism, involving glycolysis, fatty acid oxidation, and autophagy, while suppressing anabolism via inhibiting protein, fatty acid, and glycogen synthesis [78,79]. Under conditions of glucose deprivation, AMPK inhibits mTORC1 activity via two mechanisms: phosphorylation of tuberous sclerosis complex (TSC) 2 at Ser-1387, which stimulates the TSC1-TSC2 complex-induced abrogation of the ability of Ras homologue enriched in brain (Rheb) to activate mTOR, and phosphorylation of Raptor at Ser-792/Ser-722. Consequently, protein synthesis is decreased, reducing ROS production, while autophagy is increased, yielding resistance of cells to ROS [80].

FOXO activation downstream of ROS empowers the resistance to oxidative stress through activating key antioxidant enzymes, such as superoxide dismoutase (SOD), catalase, and sestrin [81]. Phosphorylation of FOXO3a by AMPK in the nucleus activates the repression of SKP2, increasing the levels of coactivator-associated arginine methyltransferase 1 (CARM1) protein, resulting in stimulation of demethylation of histone H3 Arg17, a nuclear event crucial in autophagy [82].

Sirtuins—the “magnificent seven” NAD^+^-dependent histone deacetylases (HDACs) implicated in metabolism and longevity—are crucial players of the response to CR [83], downstream of excessive ROS levels [64]. Sirtuin 1 (SIRT1) shifts FOXO-dependent responses from apoptosis to cell cycle arrest and stress resistance [84]. Sirtuin 3 (SIRT3) stimulates fatty acid oxidation and oxidative phosphorylation and stimulates the deacetylation of isocitrate dehydrogenase 2 (IDH2), an antioxidant enzyme capable of generating NADPH from oxidative decarboxylation of isocitrate to αketoglutarate. Increased NADPH, in turn, results in increased mitochondrial ratio of reduced glutathione (GSH) to oxidized Glutathione disulfide (GSSG), an alteration critical for detoxifying ROS [85]. An additional mitochondrial antioxidant enzyme activated by SIRT3 is the SOD2. The SIRT3-mediated deacetylation of two critical lysines (K53 and K89) of SOD2 facilitates the trapping of the negatively charged superoxide [86].

Beyond glucose deprivation, another mechanism increasing ROS levels is the AA starvation, resulting in activation of GCN2. GCN2 couples the AA sensing with the control of clearance of oxidized proteins and the recycling of the damaged mitochondria through autophagy [87].

The ROS-induced signaling cascades empower proliferation and survival of normal cells despite reduced nutrient availability. However, cancer cells may harness these signaling pathways to fulfill their bioenergetic and anabolic demands and continue to proliferate and survive.

Indeed, in cancer cells, limited glucose sources impair glycolysis, decrease glycolysis-based NADPH production due to reduced utilization of the pentose phosphate pathway [88,89,90,91], and shift the metabolism from glycolysis to oxidative phosphorylation (OXPHOS) (“anti-Warburg effect”), leading to ROS overload [92,93,94,95]. Cancer cells show higher ROS levels than normal cells due to dysfunctional mitochondria, oncogene activation, and impaired antioxidant defense [75]. ROS overload fosters progression of cancer by providing to cancer cells resistance to metabolic stress. For instance, activation of AMPK-mediated signaling cascades promotes survival of cancer cells via: (a) alleviation of the glucose deprivation-induced NADPH depletion through decreased fatty acid synthesis and increased fatty acid oxidation [96]; and (b) activation of the p38/proliferator-activated receptor gamma coactivator 1-alpha (PGC-1α), leading to increased mitochondrial biogenesis, OXPHOS, and ATP generation [97,98,99]. Moreover, FOXO activation downstream of AMPK facilitates the adaptation of cancer cells to nutrient deprivation via multiple mechanisms, including: (i) stimulating autophagy via expression of autophagy-related genes, such as autophagy-related gene (ATG)6, ATG7, ATG12; and (ii) supply of fatty acid and AA that are consumed by mitochondria OXPHOS [81,100].

**Figure 3:**
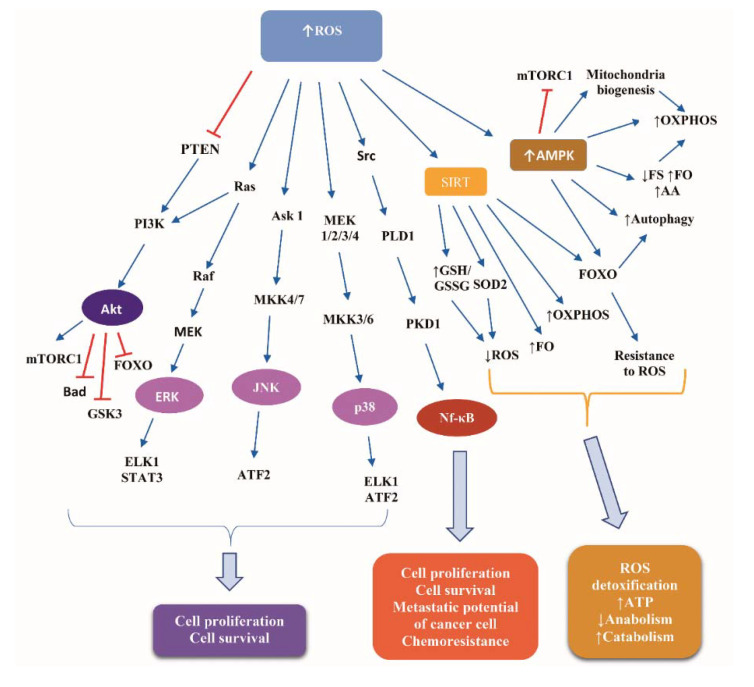
Signaling cascades downstream of reactive oxygen species in normal and cancer cells. Abbreviations: Akt, Protein kinase B; AMPK, AMP activated protein kinase; Ask 1, apoptosis signaling kinase 1; ATF2, activating transcription factor 2; ELK1, ETS Like-1 protein; ERK, extracellular signal-regulated kinases; FO, fatty acids oxidation; FOXO, Forkhead box protein; FS, fatty acids synthesis; GSH, reduced glutathione; GSK3, glucogen synthase kinase 3; GSSG, oxidized Glutathione disulfide; JNK, Janus kinase; MEK, Mitogen-activated protein kinase kinase; MKK, Mitogen-activated Protein Kinase Kinase; mTORC1, mammalian target of rapamycin complex 1; Nf-κB, nuclear factor kappa B; OXPHOS, oxidative phosphorylation; PI3K, phosphoinositide 3-kinase; PKD1, polycystic kidney disease 1; PLD1, Phospholipase D1; PTEN, phosphatase and tensin homolog; ROS, reactive oxygen species; SIRT, Sirtuin; SOD2, superoxide dismoutase; and STAT3, signal transducer and activator of transcription protein 3. Arrow ↑: increase; arrow ↓: decrease.

Figure 3 illustrates the basic signaling cascades activated in response to ROS accumulation in normal and cancer cells.

Additional tumor-promoting functions of ROS are the genomic instability, the stimulation of EMT, motility, and angiogenesis. Moreover, the oxidative stress can impair effector immune cell functions, favoring tumor-promoting immune subsets, such as myeloid-derived suppressor cells (MDSCs), therefore enhancing tumor progression [77,101].

On the other hand, excess ROS levels stimulate the death, the senescence, and the cell cycle arrest of cancer cells. Accordingly, ROS act as a double-edged sword for cancer cells [102].

To date, there are no well-established molecular mechanisms supporting a differential response to ROS signaling between normal and cancer cells. The prevailing hypothesis is that the biological outcome of ROS signaling depends on the intracellular level of ROS, the latter being regulated by a finely tuned balance between ROS production and scavenging. ROS are implicated in a wide array of signaling pathways, orchestrating cell responses to a variety of stress stimuli. Most of these pathways are common among normal and cancer cells, as depicted in Figure 3. ROS-mediated intracellular signaling transduction regulates the cell cycle and the activity of crucial transcription factors (e.g., FOXO, Nrf2, VEGF, and VEGF-R), empowering not only normal cells but also cancer cells to thrive in unfavorable conditions, such as hypoxic microenvironment [102]. ROS also trigger the release of calcium from cellular stores, activating kinases, such as protein kinase C (PKC), thereby stimulating the proliferation of normal and cancer cells. Additionally, ROS in cancer cells activate histone deacetylases (HDACs) and have a dual impact on DNA methyltransferases (DNMT), controlling the expression of oncogenes and tumor suppressor genes, such as N-Ras, K-Ras, c-Myc, and p53. Oxidized DNA bases in cancer cells trigger mutations and engage repair genes. ROS levels in cancer cells have been demonstrated to be higher than those of their normal counterparts. Accordingly, the ROS-sensitive signaling pathways are persistently stimulated in cancer cells, promoting cell transformation, genome instability, uncontrolled proliferation, carcinogenesis, EMT, and metastasis. In that respect, ROS can be proven “the Achilles heel” of cancer cells [102], rationalizing the attempt to develop anticancer strategies aiming to lower ROS levels, thereby counteracting cellular transformation, mainly by “depriving transformed cells by fuel” [102].

On the other hand, high levels of ROS can kill even redox-adapted cancer cells, mainly activating three types of programmed cell death (apoptosis, autophagy, and ferroptosis). Consequently, nuclear ROS have been suggested as the “Trojan horse” to induce DNA damage. Several anticancer strategies, including chemotherapy, molecular targeted therapies, and radiation therapy, increase ROS levels to counteract the redox adaptation of cancer cells [102]. Overall, cancer cells appear to act as “pirates” leveraging ROS for their own profit, but further increase of ROS levels can have a detrimental impact on cancer cells. The biological outcome of ROS signaling appears to depend not only on their relative concentration but also on their location. Indeed, mitochondrial ROS have been implicated in promotion of cell death, while ROS generated by the NOX family of NADPH oxidase have been credited with stimulation of cell proliferation and migration. More studies are needed to identify the determinants of the fate of cancer cells in the setting of the fasting-induced ROS signaling as well as the factors that differentiate the fate of cancer cells from that of normal cells in the same context.

## 4. Fasting Versus CR

Fasting and CR share a similar metabolic sequalae with a few subtle, but critical, differences. Fasting compared to long-term CR causes a more profound decrease in insulin (90% versus 40%, respectively) and blood glucose (50% versus 25%, respectively). The increase of utilization of protein and lipids induced by fasting is more pronounced than that induced by CR. The reduction in blood IGF-I caused in mice and humans by fasting (75%) is superior to that caused by CR (25% and 0%, in mice and in humans, respectively). In humans, CR reduces IGF-1 only when combined with protein restriction. A less intense increase in IGFBP1 occurs in fasting (≈11-fold) compared to that observed in CR (≈20-fold); nevertheless, fasting induces a 90% decrease of GH, while CR induces a 50-fold increase of GH. The difference in time required to reach the desired stress-resistant state following fasting and CR is remarkable: 2–3 days and weeks to months, respectively. FMD have been demonstrated to result in alterations of the serum levels of IGF-I, IGFBP1, glucose, and ketone bodies reminiscent of those observed in fasting [22].

A prerequisite for implementing nutrient deprivation in oncology is its safety. Despite some safety issues concerning CR, such as loss of weight, impairment of wound healing and immunological response [23], decreased fertility [103], decreased bone mineral density [104], and loss of grey matter affecting the cerebrum [105], the safety and feasibility of fasting is supported by numerous clinical studies [106,107,108,109,110,111]. Recently, FMD for three days prior to and during neoadjuvant chemotherapy as adjunct to neoadjuvant chemotherapy in women with early breast cancer in a phase II/III clinical trial (NCT02126449) showed similar grade III/IV toxicity with normal diet (75.4% for FMD group and 65.6% for control group). No significant difference was observed in the percentage of patients who discontinued chemotherapy or in terms of quality of life (QoL), either global QoL (*p*  =  0.841) or overall distress (*p*  =  0.674) [106]. In another clinical trial (NCT00936364), fasting for 24h or 48h prior to chemotherapy or 72 h (divided to 48 h prior to and 24 h post chemotherapy) was safe in patients receiving platinum-based chemotherapy for the treatment of urothelial, breast, uterine, and ovarian cancer and non-small-cell lung carcinoma [108]. Fasting-related toxicities—mainly fatigue, headache, and dizziness—were  ≤ grade 2. All patients, except one, recovered from any fasting-related weight loss prior to next chemotherapy cycle. No evidence of malnutrition was reported, but prealbumin data were not available for all subjects [108]. Taken together, available data lend support to fasting and FMDs.

## 5. Fasting-Induced Increase of the Tolerability of Chemotherapy

Although the quest for food sufficiency is implicit in human nature, fasting has been conceived as an evolutionary driving force, favoring the selection of organisms capable of resisting the challenging conditions that often accompany food deprivation, such as heat, cold, and ultraviolet radiation [112]. In that respect, fasting has been suggested as a strategy to mitigate the toxicity of chemotherapy [113], given its cellular protective effect.

### 5.1. Lessons from Nonclinical Data

Nonclinical data indicate the cellular protective effect of fasting ascribed to induction of rejuvenation in vascular, endocrine, immune, and nervous system. Short-term fasting has been shown to provide resistance to renal ischemia-reperfusion injury in mice, ameliorating insulin sensitivity, stimulating antioxidant defense, reducing inflammation, and attenuating the insulin/IGF-1 signaling [114]. In rats, fasting of donors prior to orthotopic liver transplantation slightly longer than overnight has been demonstrated to counteract the ischemia-reperfusion injury, via upregulation of heat shock proteins (HSPs) and Heme oxygenase 1 (HO1) [115]. Short-term dietary restriction (DR) prior to cardiovascular surgery provides neuroprotection to rat models of focal stroke, ascribed to regulation of innate immunity via elevation of circulating neutrophil chemoattractant C-X-C motif ligand 1 (CXCL1) prior to ischemia and suppression of striatal proinflammatory markers, such as tumor necrosis factor α (TNFa), TNFa receptor, and the downstream effector intercellular adhesion molecule-1 (ICAM-1), after reperfusion [116]. Furthermore, upregulation of neurotrophic and growth factors, such as the brain-derived neurotrophic factor (BDNF), induced by long-term DR has been shown to reduce neuronal injury after ischemia [117]. FMD cycles in old mice enhanced the cognitive performance via stimulating hippocampal neurogenesis, reducing IGF-1 and PKA signaling, and elevating Neurogenic differentiation 1 (NeuroD1), a transcription factor implicated, among others, in endocrine development of pancreatic islet cells [118]. Several mouse models have shown that the induction of autophagy, sirtuins, and proregenerative transcriptional factors mediate the protective effect of fasting [7]. A dietary restriction regimen in adult rats has been shown to decrease brain damage and ameliorate behavioral outcome in a middle cerebral artery occlusion-reperfusion (MCAO-R) stroke model [119].

Consolidating the “differential stress resistance” hypothesis, fasting combined with chemotherapy has been consistently shown to protect normal cells, but not cancer cells, against the toxicity of chemotherapy [7]. The underlying mechanisms have been suggested to be the fasting-induced reduction of IGF-I and glucose levels and the downregulation of downstream effectors [7]. A plausible explanation of the differential protective effect of fasting against chemotherapy is the attenuation of the Ras/MAPK and PI3K/Akt pathways downstream of decreased IGF-1 in normal cells contrary to the oncogene-driven constitutive activation of these pathways in cancer cells [120]. Indeed, LID mice with a conditional hepatic igf-1 gene knockout and a 70% to 80% reduction in circulating IGF-I levels, reminiscent of the reduction observed in a 72 h fasted mice, showed enhanced stress resistance against cyclophosphamide, 5-fluorouracil (5-FU), and doxorubicin. Long-term survival was observed in 60% of melanoma-bearing LID mice treated with doxorubicin, while all control mice died of either metastases or chemotherapy toxicity. Moreover, reducing IGF-I signaling protected primary glia, but not glioma cells, against cyclophosphamide and mouse embryonic fibroblasts against doxorubicin [120].

Data from both invertebrate and vertebrate animal models show that inhibition of IGF-1 signaling is an evolutionarily conserved component of longevity, yielding disease-free longer periods and alleviation of specific age-related diseases [121]. Mutations inactivating the signal transduction proteins downstream of IGF-1, such as the Ras proteins and the protein kinase Akt, have been demonstrated to provide resistance to oxidative stress in a wide array of organisms ranging from yeast to mammalians [122,123]. In fact, starvation and/or or inactivating mutations of the oncogene homolog RAS2^val19^ in yeast yields an up to 1000-fold increase in resistance to oxidative stress or chemotherapy that is prevented by overexpression/constitutive activation of Ras [124].

Notably, the gene expression signatures induced by CR resemble the ones encountered in long-lived dwarf mice carrying mutations that suppress the GH and the IGF-I signaling (Prop1^df/df^, Pit1^dw/dw^, Ghrhr^lit/lit^, and GHR-KO), particularly the Ghrhr^lit/lit^ mutation [125].

On the contrary, most human cancers bear mutations that activate IGF-1R, RAS, PI3KCA or Akt, or inactivate PTEN [126].

Additional mechanisms underlying the protective effect of fasting against chemotherapy have been described. In a rat model, 40 days of a 35% CR led to 100% protection from doxorubicin-related cardiotoxicity and death via: (i) lowering oxidative stress; (ii) induction of cardiac peroxisome proliferators activated receptor-alpha (PPAR-α) and plasma adiponectin that increased cardiac fatty acid oxidation and mitochondrial AMPK alpha2 protein kinase, resulting in 51% higher cardiac ATP levels; and (iii) upregulation of the cardioprotective Janus Kinase (JAK)/STAT3 pathway [127].

In mice, reduction of PKA activates the AMPK, which in turn activates the conserved zinc finger stress-resistance mediator EGR1 (Msn2/4 in yeast), a key transcription factor stimulating development, proliferation, and DNA repair, and apoptosis has been shown to lead not only to tumor suppression but also to protection of cardiomyocytes from doxorubicin-induced toxicity [128].

Prolonged fasting in mice led to decrease of DNA damage caused by cyclophosphamide in leukocytes and bone marrow cells and endowed hematopoietic cells with protection against chemotoxicity due to enhancement of self-renewal and regeneration [129].

Starvation combined with cisplatin has been shown in vitro to protect normal cells, promoting complete arrest of cellular proliferation mediated by p53/p21 activation in AMPK-dependent and ATM-independent manner [130]. In that respect, considering that cancer cells show no sensitivity to cell cycle inhibitors, the fasting-induced selective protection of normal cells appears rational [131].

Another interesting mechanism underlying the beneficial effect of fasting against chemotherapy is provided by a mouse model bearing colon carcinoma treated with irinotecan. In this model, preconditioning by fasting (PBF) was shown to alter the transcriptional response in the liver of mice, leading to diminished cellular injury and increased stress resistance, and alter the hepatic metabolism of irinotecan as well. Interestingly, the protective effect against the toxicity of irinotecan was not observed in the tumor tissues [132].

In a mouse model of spontaneously developed colorectal cancer, fasting for three days provided protection against the toxicity of irinotecan, while the antitumor activity of irinotecan was preserved [133].

In a mouse model of doxorubicin-induced acute cardiotoxicity, starvation for 48 h before injection of doxorubicin was demonstrated to induce autophagy by releasing the AMPK and the autophagy-initiating kinase unc-51-like kinase 1 (ULK1) from the inhibitory effect of doxorubicin [134].

Short-term starvation (STS) for up to 60 h has been shown to protect the CD-1 mice against the toxicity of doxorubicin. The combination of short-term 50% CR with either severe protein-deficiency or ketogenic diets ameliorated the resistance to the toxicity of doxorubicin in a way similar to the standard 50% CR, but the result was lower than that observed with STS [135].

Fasting for 24 h prior to treatment decreased the toxicity of etoposide in the small intestine (SI) via preservation of SI stem cell viability as well as SI architecture and barrier function [136].

Very recently, FMD was shown to reduce the tamoxifen-induced endometrial hyperplasia in mouse models of hormone receptor positive (HR+) breast cancer. Administration of tamoxifen combined with fasting or FMD led to smaller size of uteri compared to the enlarged uteri of mice treated with tamoxifen alone. The fasting-or FMD-induced counteraction of the tamoxifen-related increase in uterus size and weight was reflected on dampened histological signs of tamoxifen-related endometrial hyperplasia, such as wide, thick endometrial villi and tufts or blebs budding from the epithelium. Underlying mechanisms were indicated as: (i) the ability of fasting or FMD to decrease the expression of Tff1 (an estrogen receptor target gene) and the levels of phosphorylated Akt in mouse uteri, while increasing the mRNA of Egr1 (gene encoding tumor suppressor epidermal growth factor 1 [EGR1]) and Pten (gene encoding PTEN), irrespective of treatment with tamoxifen; and (ii) increase in PTEN and EGR1 proteins in uterus in response to FMD [137].

Table 2 summarizes the available nonclinical data concerning the protective effect of fasting against the oxidative stress and the toxicity of chemotherapy.

### 5.2. Lessons from Clinical Data

Accumulating clinical data sustain the potential of fasting to mitigate the toxicity of chemotherapy.

Recently, de Groot et al. demonstrated for the first time that FMD for three days before and during neoadjuvant chemotherapy protects against chemotoxicity in the human epidermal growth factor receptor 2 (HER2)-negative stage II/III breast cancer patients (NCT02126449). FMD significantly inhibited the chemotherapy-induced DNA damage in T-lymphocytes. DNA damage evaluated by γ-H2AX intensity in CD45+ CD3+ T-lymphocytes after chemotherapy was significantly less increased in patients with FMD compared to control group (*p* = 0.045) [106].

A landmark case series comprising 10 patients with a variety of malignancies who voluntarily fasted prior to (48–140 h) and/or following (5–56 h) chemotherapy demonstrated that short-term fasting combined with chemotherapy may alleviate the side effects of chemotherapy [109].

A randomized-controlled pilot trial (NCT01304251) compared the toxicity of docetaxel, doxorubicin, and cyclophosphamide in HER2 negative breast cancer patients who followed STF for 48 h (24 h before and after chemotherapy) compared to patients with healthy nutrition. Chemotherapy-induced DNA damage in peripheral blood mononuclear cells (PBMCs) was assessed by measurement of phosphorylation of H2AX through flow cytometry. Seven out of a total of 13 patients were randomized to the STF arm. STF showed good tolerance. A significant increase of mean erythrocyte-and thrombocyte counts seven days post-chemotherapy was observed (*p* = 0.007, 95% CI 0.106–0.638 and *p* = 0.00007, 95% CI 38.7–104, respectively) in the STF group compared to the nonSTF group. No difference concerning nonhematological toxicity was observed between the groups. Levels of γ-H2AX were significantly increased 30 min post-chemotherapy in CD45+ CD3− cells in non-STF, but not in STF patients, indicating that STF induces the DNA double-strand break repair in PBMCs after chemotherapy [107].

A protective effect of fasting for ≥48 h against the toxicity of platinum-based chemotherapy was observed in the first fasting dose-escalation study (NCT00936364). The COMET assay evaluating the oxidative stress in leukocytes revealed decreased DNA damage in leukocytes of patients fasting for ≥48 h (*p* = 0.08). Fasting for 48 h or 72 h led to decrease in Olive tail moment (live moments indicative of DNA damage in peripheral blood mononuclear cell), while fasting for 24 h was related to increased DNA damage. There was a nonsignificant trend toward less grade 3 or 4 neutropenia in the 48 h and 72 h fasting cohorts compared to the 24 h fasting cohort (*p* = 0.17). Differences in the changes of the serum levels of biomarkers such as IGF-1, insulin, and beta-hydroxybutyrate, could evaluate differences in chemotherapy toxicity among subgroups of fasting patients. Given the absence of a control group following a regular diet, this study could not explore whether fasting reduces side effects of chemotherapy [108]. A protective effect against chemotherapy-related myelosuppression was reflected on lower rates of grade 3 or 4 neutropenia in the 48 h and 72 h fasting cohorts and of grade 1 and 2 thrombocytopenia [108]. In the same study, the lower rate of neuropathy in the 48 h and 72 h fasting cohorts is remarkable, considering that these cohorts included greater number of taxane-treated patients [108].

The first clinical study designed to explore the effects of STF on quality of life (QoL), fatigue, and well-being during chemotherapy (NCT01954836) showed the feasibility of STF and its beneficial effects on QoL, well-being, and fatigue. This study enrolled thirty-four women with breast cancer and ovarian cancer randomized to a 60 h-fasting period STF (36 h before and 24 h after chemotherapy in the first half of chemotherapies followed by normocaloric diet (group A; *n* = 18) or vice versa (group B; *n* = 16). The chemotherapy-induced reduction of QoL was less than the Minimally Important Difference (MID; Functional Assessment of Cancer Therapy-General (FACT-G©) FACT-G = 5) for the STF periods but greater than the MID for nonfasted periods. The mean chemotherapy-induced deterioration of total FACIT-F of nonfasted periods was higher than that of fasted periods in both groups. STF did not induce weight loss and was associated with only minor adverse effects. It also led to a better tolerance to chemotherapy with less compromised QoL and reduced fatigue after chemotherapy [110].

Preliminary results from a Phase I clinical trial revealed that 72 h (48 h before and 24 h after chemotherapy) but not 24 h of prolonged fasting in patients undergoing two cycles of platinum-based doublet chemotherapy were associated with normal lymphocyte counts and maintenance of a normal lineage balance in white blood cells (WBCs) [129]. Fasting attenuated the chemotherapy-related immunosuppression and mortality and compensated for the age-dependent myeloid-bias in mice. Deficiencies in either IGF-1 or PKA were implicated in the proregenerative effects of fasting on stem cells.

## 6. Fasting-Induced Increase of the Efficacy of Chemotherapy

### 6.1. Lessons from Nonclinical Data

There is ample nonclinical evidence indicating that combination of fasting with chemotherapy increases the efficacy of chemotherapy, exemplifying the hypothesis of “differential stress sensitization”. To evade the chemotherapy-induced cytotoxicity, cancer cells use a multitude of strategies acquired via genetic and epigenetic alterations, most of which are interrelated with the metabolic rewiring of cancer cells [138,139,140].

Very recently, Caffa et al. demonstrated that periodic fasting or a FMD potentiates the anticancer activity of the endocrine treatment (ET) with tamoxifen and fulvestrant in vitro, in HR+ /HER2—breast cancer cell lines, and in vivo, in mouse xenografts of HR+ breast cancer cell lines and in human HR+ breast cancer organoids. The FMD-induced enhancement of the anticancer activity of ET was shown to be mediated by reduction in growth-promoting factors, namely circulating insulin, IGF-1, and leptin. Fasting/FMD and ET in breast cancer cells were shown to cooperate to reduce the Akt-mediated inhibition of tumor suppressor EGR1 (a well-established enhancer of PTEN expression), thereby increasing PTEN levels and reinforcing Akt inhibition. The upregulation of PTEN and the attendant attenuated Akt activity induced by combined ET and STS were shown to activate the AMPK, leading to reduced mTOR activity, attenuating the pro-proliferative effect of estrogen in breast cancer cells. Combined FMD and ET were demonstrated to downregulate the cyclin D1 (CCND)1 via EGR1 upregulation and Akt inhibition and thus exert an additive anticancer effect to cyclin-dependent kinase 4/6 (CDK4/6) inhibitor palbociclib or revert acquired resistance to fulvestrant plus palbociclib. Taken together, the authors suggested that the synergistic effect of fasting or FMD with ET is, at least partially, attributed to inhibition of the pro-proliferative crosstalk between insulin, IGF-1, leptin, and estrogen. The insulin-, the IGF-1-, and the leptin-induced signaling cascades converge on activation of PI3K/AKT-mTOR pathway, which stimulates cancer cell proliferation and survival, the latter being also stimulated by the estrogen-induced CDK4/6. Accordingly, inhibition of insulin, IGF-1, and leptin by fasting can co-operate with blockade of estrogen and CDK4/6 to abrogate cancer cell proliferation and survival [137]. However, the authors postulated that the potentiation of ET by fasting or FMD may implicate additional mediators, such as TNF and IL-1β [137].

In another recent study, combining CR with cisplatin or docetaxel was shown to downregulate IGF-1R and insulin receptor signaling pathways and decrease the lung metastatic burden in a triple negative breast cancer mouse model. Notably, this combination proved to counteract the chemotherapy-induced inflammatory milieu, a status incriminated for resistance to chemotherapy [141].

The combination of cisplatin with serum starvation in vitro has been demonstrated to sensitize cancer cells to cisplatin via stimulation of ATM/Checkpoint kinase 2 (Chk2)/p53 signaling pathway [130].

Cycles of starvation have been shown to exert an inhibitory effect on the growth of different tumors equal to that of doxorubicin or cyclophosphamide. Combined with either of these chemotherapeutic agents, starvation led to a more intense delay of progression of melanoma, glioma, and breast cancer cells compared to chemotherapy alone. In mouse models of neuroblastoma, fasting cycles in combination with chemotherapy resulted in long-term cancer-free survival, an outcome not observed after either treatment alone [23].

An interesting hypothesis underlying the fasting-induced sensitization of 4T1 breast cancer cells to chemotherapy is that the decrease of glucose, IGF-1, and other progrowth signals dictates increased consumption of energy as well as generation of ROS due to paradoxical activation of the AKT/S6K, partially via the AMPK-mTORC1 energy-sensing pathways malignant cells. Consequently, a status of oxidative stress is established, acting synergistically with chemotherapy to induce DNA damage. Moreover, the increased cancer cell death following combination of fasting and chemotherapy was in part ascribed to enhanced apoptosis due to activation of caspase 3 [23]. In fact, the combination of glucose restriction with a chemotherapeutic agent acted synergistically to result in a 20-fold increase in DNA damage in breast cancer and melanoma cells. In GL26 glioma cells, reduced glucose concentrations had an additive effect with doxorubicin on DNA damage [23].

Short-term starvation has been shown to act in synergy with chemotherapy to ameliorate the efficacy of the latter in an intracranial model of glioma. In vitro, glucose restriction sensitized glioma cells, but not primary glia to temozolomide [142].

A 48 h STS combined with oxaliplatin has been demonstrated to potentiate the effects of oxaliplatin, suppressing colon carcinoma growth and glucose uptake both in vitro and in vivo models, halting the progression of CT26 (undifferentiated colon carcinoma cell line) colorectal tumors. STS, via reducing the availability and the consumption of glucose, promoted a switch from aerobic glycolysis (Warburg effect) to mitochondrial OXPHOSP increasing both Complex I and Complex II-dependent O2 consumption. This anti-Warburg effect led to reduced ATP synthesis, increased ROS production, and decreased cellular redox potential, thereby promoting ROS-induced apoptosis, amplifying the toxicity and the DNA damage-dependent proapoptotic effect of OXPHOSP [143].

Another potential mechanism underlying the fasting-dependent differential stress sensitization is the enhancement of the antitumor immunity. CR mimetics enhance the immunosurveillance against transplantable, carcinogen-induced or genetically engineered cancers through induction of autophagy, which alters the metabolism of extracellular ATP, increasing the immunostimulatory ATP, which in turn stimulates antitumor immune responses mediated by cytotoxic T lymphocytes (CTLs). In parallel, autophagy reduces the adenosine-dependent recruitment of immunosuppressive regulatory T cells into the tumor bed. Given that the chemotherapy-induced attenuation of tumor growth depends entirely on CTLs, the combination of autophagy inducers with chemotherapeutic agents can amplify the antitumor immune response [144]. Indeed, STF or treatment with the CRM hydroxycitrate in mice has been shown to enhance the antitumor immunosurveillance by depletion of regulatory T cells in the setting of autophagy-competent, but not autophagy-deficient, mutant KRAS-induced lung cancers [145].

Data derived from breast cancer and melanoma indicate that fasting cycles combined with chemotherapy can reinforce the T cell-dependent targeted killing of cancer cells both by enhancing the CD8(+)-dependent tumor cytotoxicity and by expanding the Common Lymphoid Progenitors. In breast cancer, the increase of the doxorubicin-dependent tumor immunogenicity via expansion of the pool of CD8+ tumor-infiltrating lymphocytes (TILs) is partially mediated by downregulation of stress-responsive enzyme HO1, which renders the breast cancer cells more susceptible to CD8+ cytotoxic T cells, possibly by counteracting the immunosuppressive effect of regulatory T cells (Treg) [146]. Moreover, it has been demonstrated that fasting-induced autophagy can reduce CD73 levels in CT26 cancer cells, decreasing the adenosine production in the extracellular environment, thereby preventing the shift of macrophages towards an immunosuppressive M2 phenotype via inactivating the JAK1/STAT3 cascade [147].

Fasting has been shown to increase the efficacy of gemcitabine in inducing death in pancreatic cancer cells compared to controls cultured in standard medium via increasing levels of equilibrative nucleoside transporter (hENT1), responsible for transporting gemcitabine across the cell membrane, and reducing ribonucleotide reductase M1 (RRM1) levels. Consequently, fasting prior to gemcitabine injection in xenograft pancreatic cancer mice led to a decrease of more than 40% in tumor growth [148].

Starvation has been shown to modify the REV1—an inhibitory binding partner of the tumor suppressor p53—by SUMO2/3, releasing p53 from the inhibitory effect of REV1, thereby enhancing the proapoptotic effect of p53 in breast cancer and melanoma cells [149]. The fasting-induced upregulation of leptin receptor and its downstream signaling through the protein PR/SETdomain 1 (PRDM1) has been demonstrated to reverse the leukemic progression of B cell acute lymphoblastic leukemia in a human xenograft model [150].

Because avid DNA repair undermines the effectiveness of chemotherapy [56], the nutrient depletion-stimulated inhibition of DNA repair, mediated by the autophagy-induced loss of the repair enzyme 8-oxoguanine DNA glycosylase (OGG1), in vitro and in vivo [151], is a potential mechanism of sensitization of cancer cells to chemotherapy.

Nevertheless, the fasting-induced sensitization of cancer cells to chemotherapy is not a consistent finding. Cycles of short-term (3 days) 50% CR did not augment the chemotherapy efficacy of cisplatin in a murine breast cancer model [135]. Table 3 summarizes the available nonclinical data indicating that fasting increases the efficacy of chemotherapy.

**Figure 4:**
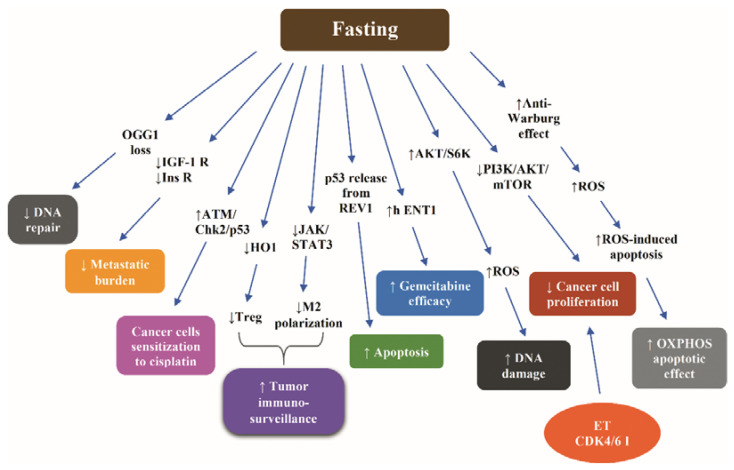
Molecular mechanisms underlying the fasting-induced increase of efficacy of chemotherapy according to nonclinical data. Abbreviations: AKT, Protein kinase B; CDK4/6 I, cyclin-dependent kinase 4/6 inhibitor; Chk2, Checkpoint kinase 2; ET, endocrine treatment; h ENT1, equilibrative nucleoside transporter; HO1, Heme oxygenase 1; IGF-1R, insulin-like growth factor 1; Ins R, insulin receptor; JAK, Janus kinase; M2, macrophages type 2; OGG1, 8-oxoguanine DNA glycosylase; PI3K, phosphoinositide 3-kinase; ROS, reactive oxygen species; S6K, S6 kinase; STAT3, signal transducer and activator of transcription protein 3; and Treg, regulatory T cells. Arrow ↑: increase; arrow ↓: decrease.

Figure 4 illustrates the most representative nonclinical data with respect to molecular mechanisms through which fasting increases the efficacy of chemotherapy.

### 6.2. Lessons from Clinical Data

Very recently, Caffa et al. reported breakthrough clinical data indicating that HR+ breast cancer is sensitive to implementation of periods of fasting, which enhance the anticancer therapy. These data concern 36 patients enrolled in either of two clinical trials, NCT03595540 (24 patients) and NCT03340935 (12 patients), designed to evaluate the safety and feasibility of periodic FMD in patients receiving active anticancer treatment. The studied patients were treated with ET (fulvestrant or tamoxifen) for a HR+ breast cancer, either as adjuvant or as palliative strategy, while one patient received fulvestrant combined with palbociclib for advanced disease. A five-day FMD (Xentigen) every four weeks was applied in the NCT03595540 trial with an average of 6.8 FMD cycles (max 14 cycles). FMD was safe, related to only grade 1–2 adverse events, mainly headache (41%) and fatigue (21%). Compared to patients enrolled in NCT03595540, the patients enrolled in NCT03340935 followed a similar, but comprising less calories, five-day FMD regimen every three to four weeks with an average of 5.5 cycles, resulting in no severe adverse events. The clinical outcomes were considered encouraging. Two patients—1 who received 10 cycles of FMD (NCT03595540) and another one who received 8 cycles of FMD (NCT03340935) presented lasting clinical control of the disease, while another one who received 8 cycles of FMD presented progression of disease after 11 months (median progression-free survival [PFS]: 9 months) (NCT03340935). One patient who received fourth-line treatment with fulvestrant and palbociclib combined with 5 FMD cycles presented disease progression after 11 months (NCT03340935). FMD led to decreased blood glucose, serum IGF-1, leptin, and C-peptide levels as opposed to increased circulating ketone bodies in all patients with HR+/HER2—breast cancer. The lower than baseline levels of leptin and IGF-1—but not insulin—persisted at least for three weeks beyond the FMD period, pointing to a carry-over anticancer effect. These clinical data built on relevant experimental data from mouse models reported in the same study, suggesting that FMD merits further evaluation as a strategy to ameliorate the efficacy of ET in patients with HR+ breast cancer (Table 3) [137].

The DIRECT trial, a multicenter randomized phase II/III trial, showed that FMD as adjunct to neoadjuvant chemotherapy for breast cancer exerts not only a protective effect against the toxicity of chemotherapy but also a beneficial effect on the radiological and pathological response to chemotherapy. The overall pCR rate was 11.7% and was similar between the two groups (10.8% in FMD group versus 12.7% in control group; OR 0.830, 95% CI 0.282–2.442, *p* = 0.735). Interestingly, the FMD group showed approximately three times more often radiologically complete or partial response, evaluated by MRI or ultrasound before surgery, compared to the control group in univariate (OR 2.886, 95% CI 1.012–8.227, *p* = 0.047) and multivariate (OR 3.168, 95% CI 1.062–9.446, *p* = 0.039) analyses. Stable or progressive disease was observed in 11.3% of patients on FMD group as opposed to 26.9% of patients in the control group.

In the per protocol (PP) analysis, the pCR rate was similar between the compliant FMD patients (13.6%) and controls (12.1%, OR 1.150, 95% CI 0.269–4.911, *p* = 0.850). However, patients with FMD showed more often Miller and Payne pathological response score 4/5 (90–100% tumor cell loss) in both univariate (OR 3.194, 95% CI 1.115–9.152, *p* = 0.031) and multivariate analyses (OR 4.109, 95% CI 1.297–13.02, *p* = 0.016) than in the control group. Furthermore, as the number of FMD cycles completed increased, more patients showed either a complete or partial radiological response to therapy (*p* for trend = 0.035) [106].

Table 4 depicts all available clinical data indicating the fasting-induced increase of the tolerability and the efficacy of chemotherapy.

## 7. Challenges and Future Perspectives in the Clinical Translation of Fasting in Oncology

The clinical translation of fasting in oncology could exemplify the notion “let thy food be thy medicine and thy medicine be thy food” attributed, though not unanimously, to Hippocrates (400 BC). To this end, current challenges should be counteracted [152].

In the era of precision medicine, implementing fasting in the subpopulation of cancer patients expected to gain the optimal profit is imperative. Future research is required to identify and harness: (a) patient-specific and tumor-specific biomarkers indicative of the sensitivity of cancer cells to fasting; (b) the mechanisms of resistance of cancer cells to fasting; (c) the distinct metabolic pathways to which each tumor is addicted; and (d) biomarkers for assessment of nutritional status during fasting.

The anticancer effect of STF per se merits further evaluation as well. So far, limited data show that fasting and FMD standalone can halt the cancer progression [23,130,142,149].

Considering that the alteration of the patients’ dietary habits is not practical, the development of fasting mimicking drugs, the repurposing of “old” drugs as CR mimetics [153], and the identification of natural products creating a CR status [154] will facilitate longer treatment periods and higher compliance. The list of compounds targeting cancer metabolism that are currently investigated in clinical trials includes metformin, aspirin, statins, rapalogs, Dichloroacetate (DCA), ADI-PEG (a pegylated [polyethylene glycol conjugated] form of the Mycoplasma-isolated arginine deiminase [ADI]), the enzyme Gossypol AZD3965, CB-839, and epigallocatechin-3-gallate (EGCG)/green tea extracts [155].

Moreover, pharmacological interventions that block simultaneously multiple key pathways of the fasting-induced signaling may increase the therapeutic efficacy of fasting. Such an intervention is the combination of fasting with tyrosine kinase inhibitors (TKI), leading to potentiation of the anticancer activity of the latter, as observed in vitro and in vivo in mice carrying human tumor xenografts. Starvation and crizotinib (TKI often prescribed for advanced nonsquamous non-small-cell lung cancer with EML4-ALK translocation) converge on the modulation of the cell cycle and DNA repair genes through activation of E2F6 (inhibitor of Inhibitor of E2F-dependent transcription) and RB1 and abrogation of the transcription factors E2F1 and E2F4 [156]. Furthermore, a synergistic effect of fasting with Sorafenib as regards inhibition of the hepatocellular carcinoma cell growth and the glucose uptake has been observed [157]. Additionally, there is emerging evidence that combining immune checkpoint inhibitors with fasting could enhance immunotherapy [158,159].

Considering that fasting modulates gut microbiota to activate the beiging of adipose tissue with a beneficial effect on metabolism [160], more studies are needed to clarify whether this strategy could potentiate the anticancer effect of fasting.

Another challenge is to explore the anticancer effect of two novel dietary interventions: the restriction of proteins or specific AA, such as methionine or tryptophan [161], and the ketogenic diets (KD) [162]. The protein restriction has been shown to thwart the tumor growth in human prostate and breast cancer models attenuating the IGF-1signaling [163]. Whether the mTORC1-independent pathways mediating the effect of methionine deprivation in metabolism [155] are implicated in cancer remains to be studied [164]. A KD is a high-fat/low-carbohydrate dietary intervention typically consisting of at least 75% fat, while carbohydrate provide a maximum 10% of energy. Restriction of carbohydrates forces the normal cells to derive energy from oxidation of fat acids and utilization of ketone bodies, an adaptation not easily adopted by cancer cells. A landmark meta-analysis demonstrated prolonged survival and decreased risk of reaching a predefined tumor volume or other sign of disease progression in mice fed on KD diet as a monotherapy compared to a diet rich in carbohydrate [165].

Given the proregenerative effect of the “ketone to glucose switch” during refeeding [111], future research should indicate the appropriate time of refeeding to preclude the detrimental additive effect of chemotherapy-induced toxicity with the acceleration of growth and proliferation signaled by cessation of fasting.

FMD merits further evaluation in terms of prevention of cancer, a perennial quest in cancer research.

A unique laboratory to investigate the consequences of intermittent fasting on health and diseases could be the Ramadan, one of the five principals of Islam creed. Ramadan is the ninth month of the Islamic calendar, established as a month of fasting, prayer, and community for Muslims worldwide. Ramadan includes a special form of fasting and alternate feasting (refeeding) with mean duration varying according to the period of the year and the latitude of the place. To date, there is a paucity of strong evidence sustaining an anticancer efficacy of Ramadan fasting [166]. Nevertheless, preliminary data indicate a multifactorial influence of Ramadan fasting on immunity [167]. Modulatory effects of fasting on the immune system have been reported, including a reduction of the activity of proinflammatory clusters of differentiation 4 (CD4) positive T helper (Th) cells and an increase in anti-inflammatory cytokines secretions like IL-4, leading to mitigation of inflammation [168]. Additional hypothetical mechanisms connecting fasting with immune system implicate the fasting-induced decrease of circulating IGF-1 and of protein kinase A (PKA) signaling, leading to modulation of hematopoietic stem cells (HSCs). The fasting-induced self-renewal, lineage regeneration, proliferation, and stress resistance of HSCs can protect the latter against the toxic effect of chemotherapy in humans. Furthermore, a fasting-induced enhancement of the phagocytic activity of macrophages has been postulated to promote the wound healing, counteracting some granulomatous infections [168]. Improved understanding of the impact of Ramadan fasting on skin anatomy, physiology, and pathophysiology may enable the establishment of evidence-based guidelines on the emerging antiaging, restorative, and anticancer role of fasting on skin [168]. Building on the effect of fasting on immunity, a therapeutic effect of Ramadan fasting on Hidradenitis Suppurativa, a systemic inflammatory disorder has been observed [169]. More studies are needed to address the anticancer efficacy of Ramadan fasting. Additionally, guidelines and standardized protocols regarding the care of cancer patients during Ramadan fasting are still lacking. Special gaps in current literature concerning cancer patients during Ramadan fasting should be further addressed, such as the quality of life, the adherence to religious worship, and the compliance with anticancer treatment [166].

A limitation to applicability of Ramadan fasting in clinical practice could be the absence of compliance to systemic drugs or even topical medications due to the fact that administration of these medications during the day may be considered as a break in the fasting. To avert any clinical and economic implications, patients should be advised to continue their treatment during fasting [168].

Finally, active clinical trials addressing fasting in oncology are anticipated to elucidate this issue [170] (Table 5). Implementation of standardized control diets, unanimously accepted definitions of interventional regimens, and close monitoring of patients, including assessment of multiple metabolic parameters, could optimize the interpretation of long-awaited clinical data [171].

## 8. Conclusions

Prescribing fasting as anticancer medicine may not be a long way ahead, provided that large randomized clinical trials consolidate its efficacy, safety, and feasibility. There is strong nonclinical evidence sustaining that fasting can increase the tolerability and efficacy of chemotherapy, awaiting consolidation in the clinics. Overall, the path forward for harnessing fasting in oncology seems to be oriented towards a personalized dietary approach guided by certified physicians.

## Figures and Tables

**Table 1:** Terminology of the most studied dietary interventions of food deprivation.

Terminology	Dietary Intervention
Fasting	Consumption of only water, for a period varying from 12 h to 3 weeks
Short term fasting (STF)	Fasting for an average of 3–5 consecutive days
Periodic fasting	Fasting repeated every 2 or more weeks
Intermittent fasting	Alternate day fasting (≥16 h) or 48 h of fasting/week
Fasting mimicking diet (FMD)	A regimen providing low calories, low amounts of proteins, and high amounts of fats. FMD provides 4600 KJ (11% protein, 46% fat, and 43% carbohydrates) for day 1 and 300 KJ (9% protein, 44% fat, and 47% carbohydrates) for days 2–5
Calorie restriction (CR)	20–40% reduction in calorie intake with reduction of all ingredients without intercepting the intake of vitamins and minerals, usually used by experts as synonym to dietary restriction (DR)

**Table 2:** Nonclinical data indicating the protective effect of fasting against chemotherapy toxicity.

Ref.	Materials	Mode of Fasting Plus CT	Outcome of Fasting Plus CT
[126]	A/J, CD-1, athymic (Nude-nu) mice	48h Starvation prior to etoposide	Protection against etoposide toxicity Improved survival of NXS2/STS/ETO compared to NXS2 mice
	Neuroblastoma (NXS2)-bearing mice		
[127]	Male Sprague-Dawley rats	35% CR prior to DXR	Protection against DXR cardiotoxicity and death
[128]	C57BL/6 mice	DXR ± STS (48 h)	Protection of cardiomyocytes against DXR toxicity by conserved PKA/AMPK/transcription factors Msn2/4 (yeast) and Egr1 (mice) pathway
[129]	C57BL/6J mice	Fasting prior to CP	Decreased DNA damage in leukocytes and bone marrow cells. Increased self-renewal and regeneration of hematopoietic cells
[130]	Primary human mesothelial SDM104 cells	Serum starvation 24 h prior to CDDP	Protection of normal cells against CDDP toxicity via complete arrest of cellular proliferation mediated by AMPK-dependent and ATM-independent p53/p21 activation
[132]	Male BALB/c mice were subcutaneously injected with C26 colon carcinoma cells	3 d Fasting prior to irinotecan	Protection of host, but not of tumor, against irinotecan toxicity by altering the transcriptional response in liver and the hepatic metabolism of irinotecan
[133]	FabplCre; Apc(15lox/+) mice spontaneously developing intestinal tumors	3 d Fasting pior to irinotecan	Protection against irinotecan toxicity
[134]	GFP-LC3 transgenic mice	48 h Fasting prior to DXR	Protection against DXR cardiotoxicity by inducing autophagy via restoring AMPK and ULK1
[135]	CD-1, BalB/C or	CR (60%, 50%, 40%, 20%, and 10% of calorie density of AIN93G) or STS (no food for up to 60 h)	Protection against DXR toxicity
	C57BL/6N mice		Protection against DXR toxicity by combination of short-term 50% CR with either severe protein-deficiency or KD similarly to 50% CR, but less than STS
[136]	Bmi1CreER/+;R26R mice	24 h Fasting prior to etoposide	Protection against etoposide toxicity in SI via preservation of SI stem cell viability, SI architecture and barrier function
	B6(Cg)-Tyrc-2 J/J,		
	Bmi1CreERT/+;Rosa26R/+ HopXCreERT/+;Rosa26R/+		
	Lgr5EGFP-IRES-CreERT2/+;Rosa26R/+,		
	Lgr5EGFP-IRES-CreERT2/+ mice		
[137]	MCF7-xenograft-bearing 6–8-week-old	Cylic FMD plus TMX	Counteraction of TMX-related increase in uterus size and weight FMD led to: ↓ expression of Tff1 (estrogen receptor target gene); ↓ levels of P-Akt in mouse uteri; ↑ mRNA of Egr1 and Pten genes ↑ PTEN and EGR1 proteins in uterus in response to FMD
	female NOD/SCIDγ mice Six-to-eight-week-old female BALB/c mice	Weekly 48-h fasting (*n* = 5) or FMD (*n* = 5) plus TMX	

Abbreviations: Akt, Protein kinase B; AL, ad libitum; AMPK, AMP-activated protein kinase; CDDP, cisplatin; CP, cyclophosphamide; CR, Calorie restriction; CT, chemotherapy; d, days; DXR, doxorubicin; Egr1 gene (EGR1 protein), epidermal growth factor 1; ETO, etoposide; FMD, fast mimicking diet; GFP-LC3, green fluorescent protein-microtubule-associated protein 1 light chain 3; h, hours; KD, ketogenic diets; P, phosphorylated; PBF, Preconditioning by fasting; PKA, protein kinase A; Pten gene (PTEN protein), Phosphatase and tensin homolog; Ref, reference; SI, small intestine; STS, short-term starvation; TMX, tamoxifen; and ULK1, unc-51-like kinase 1. Arrow ↑: increase; arrow ↓: decrease

**Table 3:** Nonclinical data indicating that fasting increases the efficacy of chemotherapy.

Ref.	Materials	Mode of Fasting Plus CT	Outcome of Fasting Plus CT
[23]	Murine and human cancer cells	24 h Starvation before and 24 h Starvation during DXR or CP	More intense delay of progression of melanoma, glioma, and breast cancer cells compared to CT alone
	Subcutaneous allografts of murine breast cancer (4T1), melanoma (B16), glioma (GL26), metastatic neuroblastoma models (NXS2, Neuro-2a), and xenografts of human neuroblastoma (ACN), breast cancer (MDA-MB-231), and ovarian cancer (OVCAR3) cell lines	48 to 60 h fasting combined with DXR or CP	
[130]	ZL55 mesothelioma cancer cells	CDDP with serum starvation	Sensitization of cancer cells, human mesothelioma xenografts, and human lung adenocarcinoma xenografts to CDDP via stimulation of ATM/Chk2/p53 signaling pathway
	Human ZL55 mesothelioma xenografts	48 h Fasting combined with CDDP	
	Human lung carcinoma A549 xenografts		
[137]	MCF7-xenograft-bearing 6–8-wk-old female NOD/SCIDγ mice	Cylic FMD plus TMX	Potentiation of FULV, TMX, palbociclib (or revertion of acquired resistance to FULV plus palbociclib via: ↓ insulin, ↓ IGF-1, ↓ leptin ↑PTEN ↓AKT ↑AMPK ↓mTOR ↓CCND1
	6-to-8-wk-old female BALB/c mice	Weekly 48 h fasting (*n* = 5) or FMD (*n* = 5) plus TMX	
[141]	Balb/c mice orthotopically injected with a syngeneic triple negative breast cancer cell line (4T1)	30% CR combined with cisplatin/docetaxel	Reversal of chemotherapy-induced inflammation Downregulation of IGF-1R and insulin receptor signaling pathways Decrease lung metastatic burden
[142]	Primary mouse glia, murine GL26, rat C6 and human U251, LN229 and A172 glioma cells	STS combined with temozolomide	Sensitization of glioma cells, but not primary glia, to TMZ Sensitization of both subcutaneous and intracranial glioma models to chemotherapy
	Mice with subcutaneous or intracranial models of GL26 glioma	48 h Starvation prior to chemotherapy	
[143]	CT26 colon carcinoma cell	48 h STS combined with OXP	Amplification of the toxicity and the DNA damage-dependent proapoptotic effect of OXP
	BALB/c mice models bearing subcutaneous CT26 colon cancer		
[146]	Murine	FMD or STS combined with FMD with DXR or CP	Additive effect on tumor suppression via T cell-dependent killing of cancer cells
	Br east cancer (4T1)	FMD combined with DXR	
	murine melanoma (B16) model		
[148]	BxPC-3, MiaPaca-2, and Panc-1 cells	Culture in combination of gemcitabine and FMM	Reinforcement of the efficacy of gemcitabine via increasing levels of h ENT1, and reducing RRM1 levels
	Pancreatic cancer xenograft mice	24 h Starvation prior to gemcitabine	
[149]	C57BL/6J mice bearing B16 melanoma tumors	48 h Fasting combined with DXR or CP	Efficacy of two fasting cycles in terms of inhibition of tumor growth equal to that of DXR or CP. Reinforcement of the efficacy of chemotherapy via induction of the proapoptotic effect of p53 due to disruption of REV1-p53 interaction

Abbreviations: CCND 1, cyclin D1; CDDP, cisplatin; Chk2, Checkpoint kinase 2; CP, cyclophosphamide; CT, chemotherapy; DXR, doxorubicin; FMM, fasting mimicking medium; FULV, fulvestrant; h ENT1, equilibrative nucleoside transporter; IGF-1R, insulin-like growth factor 1 receptor; OXP, Oxaliplatin; RRM1; ribonucleotide reductase M1; TMX, tamoxifen; TMZ, temozolomide; and wk, week.

**Table 4:** Clinical data indicating the fasting-induced increase of the tolerability and the efficacy of chemotherapy.

[Ref] Study Type (Clinical Trials Gov. Identifier)	Patients/Methods	Outcome of Fasting Plus CT
[106] Phase II/III randomized trial (NCT02126449)	131 patients with HER2-negative stage II/III breast cancer (no diabetes and BMI > 18 kg m^−2^) were randomized to receive either FMD or regular diet for 3 d prior to and during neoadjuvant CT	⮚ FMD versus control group: Similar grade III/IV toxicity (75.4% versus 65.6%, respectively; no grade V toxicity) Similar percentage of patients discontinuing CT (27.7% vs. 23.8%, respectively; *p* = 0.580) Similar QoL ⮚ FMD group: No need for dexamethasone before CT Significant inhibition of CT-induced DNA damage in T-lymphocytes by FMD (*p* = 0.045) ⮚ Overall pCR rate: 11.7% (ITT analysis. ⮚ FMD group vs. control group: pCR: 10.8% versus 12.7% pCR: 13.6% versus 12.1% More often Miller and Payne pathological response 4/5 3 times more often radiologically complete or partial response Proportion of patients with stable or progressive disease: 11.3% vs. 26.9%.
[107] Randomized-controlled pilot trial (NCT01304251)	HER2 negative breast cancer patients on 48 h STF (24 h before and after CT) were compared to patients on healthy nutrition	⮚ Good tolerance of STF ⮚ STF induces the DNA double-strand break repair in PBMCs post CT ⮚ STF group compared to the non-STF group: Significant increase of mean erythrocyte-and thrombocyte counts 7 d post-CT No difference of non-hematological toxicity
[108] Partially randomized clinical trial (NCT00936364)	Cancer patients ^a^ on platinum-based CT fasted for 24 h or 48 h prior CT or 72 h (48 h prior to and 24 h post CT)	⮚ Fasting led to: Toxicities of grade ≤ 2 (fatigue, headache, and dizziness) Recovery from any of the fasting-related weight loss prior to next CT cycle for all patients except one No evidence of malnutrition Decreased DNA damage in leukocytes for fasting for ≥48 h (*p* = 0.08) Varying changes of serum biomarkers reflecting distinct CT toxicity ⮚ 48 h and 72 h fasting cohorts vs. 24 h cohort: Nonsignificant trend toward less grade 3 or 4 neutropenia (*p* = 0.17) Less grade 1 and 2 thrombocytopenia Lower rate of neuropathy
[109] Case series	10 cancer patients voluntarily fasted prior to (48–140 h) and/or following (5–56 h) CT	⮚ STF plus CT may alleviate the CT toxicity ⮚ Side effects of fasting: hunger and lightheadedness ⮚ No compromise of CT efficacy
[110] Individually randomized cross-over trial (NCT01954836)	34 women with breast cancer or ovarian cancer were randomized to a 60 h STF (36 h before and 24 h after CT) in the first half of CT followed by normocaloric diet (group A; *n* = 18) or vice versa (group B; *n* = 16)	⮚ CT-induced reduction of QoL < MID (FACT-G = 5) for the STF periods but > MID for non-fasted periods ⮚ Mean CT-induced deterioration of total FACIT-F of nonfasted periods higher than that of fasted periods in both groups ⮚ STF led to: No weight loss Minor adverse effects Better tolerance to CT Less compromised QoL and reduced fatigue within 8 d after chemotherapy
[129] Phase I clinical trial (NCT00936364)	Patients with malignant solid tumors were treated with platinum-based doublet chemotherapy combined with 72 h fasting (48 h before and 24 h after CT)	⮚ Fasting led to: Normal lymphocyte counts and normal lineage balance in WBCs Attenuation of the CT-related immunosuppression and mortality Reversal of age-dependent myeloid-bias in mice Proregenerative effects on stem cells due to IGF-1 or PKA deficiencies
[137] Interventional clinical trials NCT03595540 (24 patients) NCT03340935 (12 patients)	35 patients with HR+ breast cancer treated with FULV or TMX, as adjuvant or as palliative strategy, and 1 patient treated with fulvestrant plus palbociclib for advanced disease followed 5 d FMD (Xentigen) Q4W (NCT03595540) (average 6.8 FMD cycles, max 14 cycles) or 5 d FMD regimen Q3-4W (average 5.5 cycles)	⮚ FMD led to: No severe adverse events Lasting clinical control of disease (2 patients) Disease progression after 11 mo (median progression-free survival [PFS]: 9 mo) (1 patient) Disease progression after 11 mo (1 patient treated with FULV plus palbociclib (CDK4/6 inhibitor) combined with 5 FMD cycles ↓blood glucose, serum IGF-1, leptin, and C-peptide levels in all patients ↑circulating ketone bodies in all patients

^a^ urothelial, breast, uterine, and ovarian cancer, and non-small-cell lung carcinoma. Abbreviations: BMI, body mass index; CDK4/6, cyclin-dependent kinase 4/6; CT, chemotherapy; d, days; h, hours; FACIT-F, functional assessment of chronic illness therapy (a 13-item questionnaire that assesses self-reported fatigue and its impact upon daily activities); FACT-G, Functional Assessment of Cancer Therapy-General; FMD fasting mimicking diet; FULV, fulvestrant; HER2, human epidermal growth factor receptor 2; HR, hormone receptor; IGF-1, insulin-like growth factor 1; ITT, intention to treat; mo, months; MID, Minimally Important Difference; PBMCs, peripheral blood mononuclear cells; PKA, protein kinase A; PP, per protocol; Q4W, every 4 weeks; Q3-4W, every 3 or 4 weeks; STF, short-term fasting; TMX, tamoxifen; and WBCs, white blood cells. Arrow ↑: increase; arrow ↓: decrease.

**Table 5:** Active clinical trials addressing fasting in oncology.

Cancer Type Clinical Trials Gov. Identifier	Arm I: Intervention	Arm II: Active Comparator	Primary Endpoint	Time Frame
Breast Cancer Prostate Cancer NCT01802346	Low-calorie diet 3 d before and 24 h after CT during the 12 wks of CT	Normal diet	Impact on toxicity and efficacy of CT Compliance Changes in plasma insulin, glucose, IGF-1, IGFBP levels	12 wks
Non-small Cell Lung Cancer NCT03700437	Chemolieve^®^ in patients on carboplatin/pemetrexed and pembrolizumab 3 d before CT/immunotherapy and on the 1st day of CT/immunotherapy for the first 4 C	Control arm: regular diet	Change in CTC Evaluation of γ-H2AΧ foci in CTCs Changes in PBMC	Screening baseline and on: C1 d 1 C2 d 1 End treatment
Prostatic Neoplasms NCT02710721	60 h-FMD (36 h before and 24 h after CT)	MD	Change of FACT-P/-Taxane/-A sum score from baseline to day 8 after each CT	Baseline 7 days after each of 6 CT (wks 1,4,7,10,13,16) 3 and 6 mo after d 0
Breast cancer Ovarian cancer NCT03162289	Intermittent fasting 60–72 h (36–48 h before and 24 h after CT)	60–72 h Vegan ^d^ 36–48 h before and 24 h after CT during the first 4 C of CT and thereafter 2 d (24 h before and after CT) vegan and sugar-restricted diet	Change of FACT-G score	Baseline day −2 and +7 at each CT (triweekly C) −2 days at each CT (weekly C) +7 after the last weekly CT 4 mo after inclusion 3 wks after end of CT 1, 2, 3 years after inclusion
Cancer Breast Cancer Colorectal Cancer NCT03595540	Monthly C of Prolon FMD (L-Nutra) in patients under active cancer treatment	NA	Feasibility of FMD Quantification of FMD-emergent adverse events	6 mo
Breast cancer Melanoma malignant NCT03454282	5-day FMD followed for 1 C (Cohorts A and B) or for 4 consecutive every-4-week C, postop.	NA	Changes in PBMCs	3 years
Glioblastoma NCT03451799	16-wk KD while on standard of care cancer treatment (Radiation + Temozolomide)	NA	Safety of KD	4 mo
Advanced LKB1-inactive Lung Adenocarcinoma NCT03709147	Every-three wks, 5-d-FMD up to 4 C in patients receiving: Metformin Hydrochloride Cisplatin Carboplatin Pemetrexed	Metformin Hydrochloride Cisplatin Carboplatin Pemetrexed	Progression-free survival	60 mo
Malignant Neoplasm Cancer NCT03340935	FMD	NA	Safety of FMD	2 years
Cancer NCT03840213	Behavioral: Filling a questionnaire and interview focused on diet	NA	N of patients who voluntarily changed eating habits or followed fasting or restrictive diet during CT	1 year
Glioblastoma Multiforme NCT01865162	KD as adjuvant for treatment-refractory glioblastoma multiforme	NA	Safety of KD	1 year
Glioblastoma Multiforme NCT02302235	KD adjunctive to standard radiation and temozolomide CT	Phase 2	Survival Time to radiological (MRI) tumor progression Incidence of TEAE	6 mo
Glioblastoma Multiforme NCT01535911	KD in adults with newly diagnosed glioblastoma while being on RT and CT	NA	Changes in brain tumor size assessed by MRI	6 wks after RT completion
Childhood cancer survivors NCT03523377	Overnight fasting (12h) after completion of therapy	NA	Measure of Glu metabolism	6 mo

^d^ A 60–72 h vegan diet with sugar restriction (36–48 h before and 24 h after CT) for the first four cycles of CT. During the rest of the CT cycles, patients will follow two days of vegan and sugar-restricted diet (24 h before and after CT). Between CT cycles a mainly vegetarian diet will be followed. Abbreviations: C, cycle(s); CT, chemotherapy; CTC(s), circulating tumor cell(s); d, day; FACT-G, Functional Assessment of Cancer Therapy—GeneraL; FACT-P Functional Assessment of Cancer Therapy-Prostate; FMD, fasting mimicking diet; Glu, glucose; h, hour; γ-H2AΧ, phosphorylated form of H2A histone family member X; IGF-1, insulin-like growth factor 1; IGFBP, IGF binding protein; KD, ketogenic diet; mo, months; MD, mediterranean diet; mo, months; MRI, magnetic resonance imaging; N, number; PBMCs, Peripheral blood mononuclear cells; RT, radiotherapy; TEAE, treatment emergent adverse events; and wk(s), week(s).

## References

[1] Moreschi C. (1909). Beziehung zwischen ernahrung and tumorwachstum. ZImmunitatsforsch.

[2] McCay C.M., Crowel M.F., Maynard L.A. (1935). The effect of retarded growth upon the length of the life span and upon the ultimate body size. J. Nutr..

[3] Kritchevsky D. (2001). Caloric restriction and cancer. J. Nutr. Sci. Vitaminol..

[4] McDonald R.B., Ramsey J.J. (2010). Honoring Clive McCay and 75 years of calorie restriction research. J. Nutr..

[5] Wilhelmi de Toledo F., Buchinger A., Burggrabe H., Hölz G., Kuhn C., Lischka E., Lischka N., Lützner H., May W., Ritzmann-Widderich M. (2013). Fasting Therapy—An Expert Panel Update of the 2002 Consensus Guidelines. Forsch. Komplementmed..

[6] Buono R., Longo V.D. (2018). Starvation, Stress Resistance, and Cancer. Trends Endocrinol. Metab..

[7] Nencioni A., Caffa I., Cortellino S., Longo V.D. (2018). Fasting and cancer: Molecular mechanisms and clinical application. Nat. Rev. Cancer.

[8] Cancer statistics. https://seer.cancer.gov/statfacts/html/all.html.

[9] Xu M., Pu Y., Weichselbaum R.R., Fu Y.X. (2016). Integrating conventional and antibody-based targeted anticancer treatment into immunotherapy. Oncogene.

[10] Laviano A., Molfino A., Fanelli F.R. (2012). Cancer-treatment toxicity: Can nutrition help?. Nat. Rev. Clin. Oncol..

[11] Costa A.S.H., Frezza C. (2017). Metabolic Reprogramming and Oncogenesis: One Hallmark, Many Organelles. Int. Rev. Cell Mol. Biol..

[12] Yoshida G.J. (2015). Metabolic reprogramming: The emerging concept and associated therapeutic strategies. J. Exp. Clin. Cancer Res..

[13] Hirschey M.D., DeBerardinis R.J., Diehl A.M.E., Drew J.E., Frezza C., Green M.F., Jones L.W., Ko Y.H., Le A., Lea M.A. (2015). Dysregulated metabolism contributes to oncogenesis. Semin. Cancer Biol..

[14] Guerra F., Arbini A.A., Moro L. (2017). Mitochondria and cancer chemoresistance. Biochim Biophys Acta Bioenerg..

[15] Caro M.M., Laviano A., Pichard C. (2007). Nutritional intervention and quality of life in adult oncology patients. Clin. Nutr..

[16] Arends J., Bachmann P., Baracos V., Barthelemy N., Bertz H., Bozzetti F., Fearon K., Hütterer E., Isenring E., Kaasa S. (2017). ESPEN guidelines on nutrition in cancer patients. Clin. Nutr..

[17] Emmons K.M., Colditz G.A. (2017). Realizing the Potential of Cancer Prevention—The Role of Implementation Science. N. Engl. J. Med..

[18] Fontana L., Partridge L., Longo V.D. (2010). Extending healthy life span—From yeast to humans. Science.

[19] O’Flanagan C.H., Smith L.A., McDonell S.B., Hursting S.D. (2017). When less may be more: Calorie restriction and response to cancer therapy. BMC Med..

[20] Mattson M.P., Longo V.D., Harvie M. (2017). Impact of intermittent fasting on health and disease processes. Ageing Res. Rev..

[21] Simone B.A., Champ C.E., Rosenberg A.L., Berger A.C., Monti D.A., Dicker A.P., Simone N.L. (2013). Selectively starving cancer cells through dietary manipulation: Methods and clinical implications. Future Oncol..

[22] Lee C., Longo V. (2011). Fasting vs. dietary restriction in cellular protection and cancer treatment: From model organisms to patients. Oncogene.

[23] Lee C., Raffaghello L., Brandhorst S., Safdie F.M., Bianchi G., Martin-Montalvo A., Pistoia V., Wei M., Hwang S., Merlino A. (2012). Fasting cycles retard growth of tumors and sensitize a range of cancer cell types to chemotherapy. Sci. Transl. Med..

[24] Varady K.A., Hellerstein M.K. (2007). Alternate-day fasting and chronic disease prevention: A review of human and animal trials. Am. J. Clin. Nutr..

[25] Goodrick C.L., Ingram D.K., Reynolds M.A., Freeman J.R., Cider N.L. (1983). Differential effects of intermittent feeding and voluntary exercise on body weight and lifespan in adult rats. J. Gerontol..

[26] Brandhorst S., Choi I.Y., Wei M., Cheng C.W., Sedrakyan S., Navarrete G., Dubeau L., Yap L.P., Park R., Vinciguerra M. (2015). A Periodic Diet that Mimics Fasting Promotes Multi-System Regeneration, Enhanced Cognitive Performance, and Healthspan. Cell Metab..

[27] Wei M., Brandhorst S., Shelehchi M., Mirzaei H., Cheng C.W., Budniak J., Groshen S., Mack W.J., Guen E., Di Biase S. (2017). Fasting- mimicking diet and markers/risk factors for aging, diabetes, cancer, and cardiovascular disease. Sci. Transl. Med..

[28] Liang Y., Liu C., Lu M., Dong Q., Wang Z., Wang Z., Xiong W., Zhang N., Zhou J., Liu Q. (2018). Calorie restriction is the most reasonable anti-ageing intervention: A meta-analysis of survival curves. Sci. Rep..

[29] Swindell W.R. (2012). Dietary restriction in rats and mice: A meta-analysis and review of the evidence for genotype-dependent effects on lifespan. Ageing Res. Rev..

[30] Nakagawa S., Lagisz M., Hector K.L., Spencer H.G. (2012). Comparative and meta-analytic insights into life extension via dietary restriction. Aging Cell.

[31] Jensen K., McClure C., Priest N.K., Hunt J. (2015). Sex-specific effects of protein and carbohydrate intake on reproduction but not lifespan in Drosophila melanogaster. Aging Cell.

[32] Lauby-Secretan B., Scoccianti C., Loomis D., Grosse Y., Bianchini F., Straif K., International Agency for Research on Cancer Handbook Working Group (2016) (2016). Body Fatness and Cancer—Viewpoint of the IARC Working Group. N. Engl. J. Med..

[33] Weindruch R., Walford R.L., Fligiel S., Guthrie D. (1986). The retardation of aging in mice by dietary restriction: Longevity, cancer, immunity and lifetime energy intake. J. Nutr..

[34] Colman R.J., Anderson R.M., Johnson S.C., Kastman E.K., Kosmatka K.J., Beasley T.M., Allison D.B., Cruzen C., Simmons H.A., Kemnitz J.W. (2009). Caloric restriction delays disease onset and mortality in rhesus monkeys. Science.

[35] Mattison J.A., Roth G.S., Beasley T.M., Tilmont E.M., Handy A.M., Herbert R.L., Longo D.L., Allison D.B., Young J.E., Bryant M. (2012). Impact of caloric restriction on health and survival in rhesus monkeys from the NIA study. Nature.

[36] Speakman J.R., Mitchell S.E. (2011). Caloric restriction. Mol. Aspects Med..

[37] Kopeina G.S., Senichkin V.V., Zhivotovsky B. (2017). Caloric restriction—A promising anti-cancer approach: From molecular mechanisms to clinical trials. Biochim. Biophys. Acta Rev. Cancer.

[38] Redman L.M., Heilbronn L.K., Martin C.K., Alfonso A., Smith S.R., Ravussin E., Pennington CALERIE Team (2007). Effect of calorie restriction with or without exercise on body composition and fat distribution. J. Clin. Endocrinol. Metab..

[39] Rochon J., Bales C.W., Ravussin E., Redman L.M., Holloszy J.O., Racette S.B., Roberts S.B., Das S.K., Romashkan S., Galan K.M. (2011). Design and conduct of the CALERIE study: Comprehensive assessment of the long-term effects of reducing intake of energy. J. Gerontol. A Biol. Sci. Med. Sci..

[40] Fontana L., Klein S. (2007). Aging, adiposity, and calorie restriction. JAMA.

[41] Lope V., Martín M., Castelló A., Ruiz A., Casas A.M., Baena-Cañada J.M., Antolín S., Ramos-Vázquez M., García-Sáenz J.Á., Muñoz M. (2019). Overeating, caloric restriction and breast cancer risk by pathologic subtype: The EPIGEICAM study. Sci. Rep..

[42] Dirx M.J., Zeegers M.P., Dagnelie P.C., van den Bogaard T., van den Brandt P.A. (2003). Energy restriction and the risk of spontaneous mammary tumors in mice: A meta-analysis. Int. J. Cancer.

[43] Lv M., Zhu X., Wang H., Wang F., Guan W. (2014). Roles of caloric restriction, ketogenic diet and intermittent fasting during initiation, progression and metastasis of cancer in animal models: A systematic review and meta-analysis. PLoS ONE.

[44] Masharani U., Gitelman S.E. (2011). Hypoglemic Disorders. Greenspan’s Basic & Clinical Endocrinology.

[45] Longo V.D., Mattson M.P. (2014). Fasting: Molecular mechanisms and clinical applications. Cell Metab..

[46] Naveed S., Aslam M., Ahmad A. (2014). Starvation based differential chemotherapy: A novel approach for cancer treatment. Oman Med. J..

[47] Gupta R., Ma Y., Wang M., Whim M.D. (2017). AgRP-Expressing Adrenal Chromaffin Cells Are Involved in the Sympathetic Response to Fasting. Endocrinology.

[48] Wang Q., Whim M.D. (2013). Stress-induced changes in adrenal neuropeptide Y expression are regulated by a negative feedback loop. J. Neurochem..

[49] Dogan S., Ray A., Cleary M.P. (2017). The influence of different calorie restriction protocols on serum pro-inflammatory cytokines, adipokines and IGF-I levels in female C57BL6 mice: Short term and long term diet effects. Meta Gene.

[50] Messaoudi I., Warner J., Fischer M., Park B., Hill B., Mattison J., Lane M.A., Roth G.S., Ingram D.K., Picker L.J. (2006). Delay of T cell senescence by caloric restriction in aged long-lived nonhuman primates. Proc. Natl. Acad. Sci. USA.

[51] Dong S., Khoo A., Wei J., Bowser R.K., Weathington N.M., Xiao S., Zhang L., Ma H., Zhao Y., Zhao J. (2014). Serum starvation regulates E-cadherin upregulation via activation of c-Src in non-small-cell lung cancer A549 cells. Am. J. Physiol. Cell Physiol..

[52] Orgel E., Mittelman S.D. (2013). The links between insulin resistance, diabetes, and cancer. Curr. Diab. Rep..

[53] Dao M.C., Sokolovska N., Brazeilles R., Affeldt S., Pelloux V., Prifti E., Chilloux J., Verger E.O., Kayser B.D., Aron-Wisnewsky J. (2019). A Data Integration Multi-Omics Approach to Study Calorie Restriction-Induced Changes in Insulin Sensitivity. Front. Physiol..

[54] Lu C., Shi Y., Wang Z., Song Z., Zhu M., Cai Q., Chen T. (2008). Serum starvation induces H2AX phosphorylation to regulate apoptosis via p38 MAPK pathway. FEBS Lett..

[55] Braun F., Bertin-Ciftci J., Gallouet A.S., Millour J., Juin P. (2011). Serum-nutrient starvation induces cell death mediated by Bax and Puma that is counteracted by p21 and unmasked by Bcl-x(L) inhibition. PLoS ONE.

[56] Zhang D., Tang B., Xie X., Xiao Y.F., Yang S.M., Zhang J.W. (2015). The interplay between DNA repair and autophagy in cancer therapy. Cancer Biol. Ther..

[57] Rodríguez-Vargas J.M., Ruiz-Magaña M.J., Ruiz-Ruiz C., Majuelos-Melguizo J., Peralta-Leal A., Rodríguez M.I., Muñoz-Gámez J.A., de Almodóvar M.R., Siles E., Rivas A.L. (2012). ROS-induced DNA damage and PARP-1 are required for optimal induction of starvation-induced autophagy. Cell Res..

[58] Huang Q., Shen H.M. (2009). To die or to live: The dual role of poly(ADP-ribose) polymerase-1 in autophagy and necrosis under oxidative stress and DNA damage. Autophagy.

[59] Dröge W. (2002). Free Radicals in the Physiological Control of Cell Function. Physiol. Rev..

[60] Santos A.L., Sinha S., Lindner A.B. (2018). The Good, the Bad, and the Ugly of ROS: New Insights on Aging and Aging-Related Diseases from Eukaryotic and Prokaryotic Model Organisms. Oxid. Med. Cell Longev..

[61] Liao Z., Damien C., Tan N. (2019). Reactive oxygen species: A volatile driver of field cancerization and metastasis. Mol. Cancer.

[62] Valko M., Leibfritz D., Moncol J., Cronin M.T., Mazur M., Telser J. (2007). Free radicals and antioxidants in normal physiological functions and human disease. Int. J. Biochem. Cell Biol..

[63] Gredilla R., Barja G. (2005). Minireview: The role of oxidative stress in relation to caloric restriction and longevity. Endocrinology.

[64] Salminen A., Kaarniranta K., Kauppinen A. (2013). Crosstalk between Oxidative Stress and SIRT1: Impact on the Aging Process. Int. J. Mol. Sci..

[65] Calabrese E.J., McCarthy M.E., Kenyon E. (1987). The occurrence of chemically induced hormesis. Health Phys..

[66] Zimmermann A., Bauer M.A., Kroemer G., Madeo F., Carmona-Gutierrez D. (2014). When less is more: Hormesis against stress and disease. Microb. Cell..

[67] Poljsak B. (2011). Strategies for reducing or preventing the generation of oxidative stress. Oxid. Med. Cell Longev..

[68] Martucci M., Ostan R., Biondi F., Bellavista E., Fabbri C., Bertarelli C., Salvioli S., Capri M., Franceschi C., Santoro A. (2017). Mediterranean diet and inflammaging within the hormesis paradigm. Nutr. Rev..

[69] Ferre P., Azzout-Marniche D., Foufelle F. (2003). AMP-activated protein kinase and hepatic genes involved in glucose metabolism. Biochem. Soc. Trans..

[70] Viollet B., Foretz M., Guigas B., Horman S., Dentin R., Bertrand L., Hue L., Andreelli F. (2006). Activation of AMP-activated protein kinase in the liver: A new strategy for the management of metabolic hepatic disorders. J. Physiol..

[71] Proud C.G. (2004). Role of mTOR signalling in the control of translation initiation and elongation by nutrients. Curr. Top Microbiol. Immunol..

[72] Anthony T.G., McDaniel B.J., Byerley R.L., McGrath B.C., Cavener D.R., McNurlan M.A., Wek R.C. (2004). Preservation of liver protein synthesis during dietary leucine deprivation occurs at the expense of skeletal muscle mass in mice deleted for eIF2 kinase GCN2. J. Biol. Chem..

[73] Dever T.E., Hinnebusch A.G. (2005). GCN2 whets the appetite for amino acids. Mol. Cell.

[74] González A., Hall M.N. (2017). Nutrient sensing and TOR signaling in yeast and mammals. EMBO J..

[75] Towle H.C. (2007). The metabolic sensor GCN2 branches out. Cell Metab..

[76] Zhang J., Wang X., Vikash V., Ye Q., Wu D., Liu Y., Dong W. (2016). ROS and ROS-Mediated Cellular Signaling. Oxid. Med. Cell Longev..

[77] Liou G.Y., Storz P. (2010). Reactive oxygen species in cancer. Free Radic. Res..

[78] Hardie D.G., Ross F.A., Hawley S.A. (2012). AMPK: A nutrient and energy sensor that maintains energy homeostasis. Nat. Rev. Mol. Cell Biol..

[79] Masharani U., German M.S. (2011). Pancreatic Hormones and Diabetes Mellitus. Greenspan’s Basic & Clinical Endocrinology.

[80] Zhao Y., Hu X., Liu Y., Dong S., Wen Z., He W., Zhang S., Huang Q., Shi M. (2017). ROS signaling under metabolic stress: Cross-talk between AMPK and AKT pathway. Mol. Cancer.

[81] Chiacchiera F., Simone C. (2010). The AMPK-FoxO3A axis as a target for cancer treatment. Cell Cycle.

[82] Shin H.J., Kim H., Oh S., Lee J.G., Kee M., Ko H.J., Kweon M.N., Won K.J., Baek S.H. (2016). AMPK-SKP2-CARM1 signalling cascade in transcriptional regulation of autophagy. Nature.

[83] Dali-Youcef N., Lagouge M., Froelich S., Koehl C., Schoonjans K., Auwerx J. (2007). Sirtuins: The ‘magnificent seven’, function, metabolism and longevity. The sirtuin family of histone deacetylases (HDACs). Ann. Med..

[84] Brunet A., Sweeney L.B., Sturgill J.F., Chua K.F., Greer P.L., Lin Y., Tran H., Ross S.E., Mostoslavsky R., Cohen H.Y. (2004). Stress-dependent regulation of FOXO transcription factors by the SIRT1 deacetylase. Science.

[85] Yu W., Dittenhafer-Reed K.E., Denu J.M. (2012). SIRT3 protein deacetylates isocitrate dehydrogenase 2 (IDH2) and regulates mitochondrial redox status. J. Biol. Chem..

[86] Qiu X., Brown K., Hirschey M.D., Verdin E., Chen D. (2010). Calorie restriction reduces oxidative stress by SIRT3-mediated SOD2 activation. Cell Metab..

[87] Ravindran R., Loebbermann J., Nakaya H.I., Khan N., Ma H., Gama L., Machiah D.K., Lawson B., Hakimpour P., Wang Y.C. (2016). The amino acid sensor GCN2 controls gut inflammation by inhibiting inflammasome activation. Nature.

[88] Ahmad I.M., Aykin-Burns N., Sim J.E., Walsh S.A., Higashikubo R., Buettner G.R., Venkataraman S., Mackey M.A., Flanagan S.W., Oberley L.W. (2005). Mitochondrial O_2_*- and H_2_O_2_ mediate glucose deprivation-induced stress in human cancer cells. J. Biol. Chem..

[89] Ward P.S., Thompson C.B. (2012). Metabolic reprogramming: A cancer hallmark even warburg did not anticipate. Cancer Cell.

[90] Patra K.C., Hay N. (2014). The pentose phosphate pathway and cancer. Trends Biochem. Sci..

[91] Harris I.S., Treloar A.E., Inoue S., Sasaki M., Gorrini C., Lee K.C., Yung K.Y., Brenner D., Knobbe-Thomsen C.B., Cox M.A. (2015). Glutathione and thioredoxin antioxidant pathways synergize to drive cancer initiation and progression. Cancer Cell.

[92] Birsoy K., Possemato R., Lorbeer F.K., Bayraktar E.C., Thiru P., Yucel B., Wang T., Chen W.W., Clish C.B., Sabatini D.M. (2014). Metabolic determinants of cancer cell sensitivity to glucose limitation and biguanides. Nature.

[93] Moley K.H., Mueckler M.M. (2000). Glucose transport and apoptosis. Apoptosis.

[94] Balaban R.S., Nemoto S., Finkel T. (2005). Mitochondria, oxidants, and aging. Cell.

[95] Harman D. (1956). Aging: A theory based on free radical and radiation chemistry. J. Gerontol..

[96] Jeon S.M., Chandel N.S., Hay N. (2012). AMPK regulates NADPH homeostasis to promote tumour cell survival during energy stress. Nature.

[97] Chaube B., MalvI P., Singh S.V., Mohammad N., Viollet B., Bhat M.K. (2015). AMPK maintains energy homeostasis and survival in cancer cells via regulating p38/PGC-1α-mediated mitochondrial biogenesis. Cell Death Discov..

[98] Suwa M., Nakano H., Kumagai S. (2003). Effects of chronic AICAR treatment on fiber composition, enzyme activity, UCP3, and PGC-1 in rat muscles. J. Appl. Physiol..

[99] Terada S., Goto M., Kato M., Kawanaka K., Shimokawa T., Tabata I. (2002). Effects of low-intensity prolonged exercise on PGC-1 mRNA expression in rat epitrochlearis muscle. Biochem. Biophys. Res. Commun..

[100] Chiacchiera F., Simone C. (2009). Inhibition of p38alpha unveils an AMPK-FoxO3A axis linking autophagy to cancer-specific metabolism. Autophagy.

[101] Ohl K., Tenbrock K. (2018). Reactive Oxygen Species as Regulators of MDSC-Mediated Immune Suppression. Front. Immunol..

[102] Perillo B., Di Donato M., Pezone A., Di Zazzo E., Giovannelli P., Galasso G., Castoria G., Migliaccio A. (2020). ROS in cancer therapy: The bright side of the moon. Exp. Mol. Med..

[103] Moatt J.P., Nakagawa S., Lagisz M., Walling C.A. (2016). The effect of dietary restriction on reproduction: A meta-analytic perspective. BMC Evol. Biol..

[104] Villareal D.T., Fontana L., Weiss E.P., Racette S.B., Steger-May K., Schechtman K.B., Klein S., Holloszy J.O. (2006). Bone mineral density response to caloric restriction-induced weight loss or exercise-induced weight loss: A randomized controlled trial. Arch. Intern. Med..

[105] Pifferi F., Terrien J., Marchal J., Dal-Pan A., Djelti F., Hardy I., Chahory S., Cordonnier N., Desquilbet L., Hurion M. (2018). Caloric restriction increases lifespan but affects brain integrity in grey mouse lemur primates. Commun. Biol..

[106] De Groot S., Lugtenberg R.T., Cohen D., Welters M.J.P., Ehsan I., Vreeswijk M., Smit V., de Graaf H., Heijns J.B., Portielje J. (2020). Fasting mimicking diet as an adjunct to neoadjuvant chemotherapy for breast cancer in the multicentre randomized phase 2 DIRECT trial. Nat. Commun..

[107] De Groot S., Vreeswijk M.P., Welters M.J., Gravesteijn G., Boei J.J., Jochems A., Houtsma D., Putter H., van der Hoeven J.J., Nortier J.W. (2015). The effects of short-term fasting on tolerance to (neo) adjuvant chemotherapy in HER2-negative breast cancer patients: A randomized pilot study. BMC Cancer.

[108] Dorff T.B., Groshen S., Garcia A., Shah M., Tsao-Wei D., Pham H., Cheng C.W., Brandhorst S., Cohen P., Wei M. (2016). Safety and feasibility of fasting in combination with platinum-based chemotherapy. BMC Cancer.

[109] Safdie F.M., Dorff T., Quinn D., Fontana L., Wei M., Lee C., Cohen P., Longo V.D. (2009). Fasting and cancer treatment in humans: A case series report. Aging.

[110] Bauersfeld S.P., Kessler C.S., Wischnewsky M., Jaensch A., Steckhan N., Stange R., Kunz B., Brückner B., Sehouli J., Michalsen A. (2018). The effects of short-term fasting on quality of life and tolerance to chemotherapy in patients with breast and ovarian cancer: A randomized cross-over pilot study. BMC Cancer.

[111] Wilhelmi de Toledo F., Grundler F., Bergouignan A., Drinda S., Michalsen A. (2019). Safety, health improvement and well-being during a 4 to 21-day fasting period in an observational study including 1422 subjects. PLoS ONE.

[112] Stone J., Mitrofanis J., Johnstone D.M., Falsini B., Bisti S., Adam P., Nuevo A.B., George-Weinstein M., Mason R., Eells J. (2018). Acquired Resilience: An Evolved System of Tissue Protection in Mammals. Dose Response.

[113] Nurgali K., Jagoe R.T., Abalo R. (2018). Editorial: Adverse Effects of Cancer Chemotherapy: Anything New to Improve Tolerance and Reduce Sequelae?. Front. Pharmacol..

[114] Mitchell J.R., Verweij M., Brand K., van de Ven M., Goemaere N., van den Engel S., Chu T., Forrer F., Müller C., de Jong M. (2009). Short-term dietary restriction and fasting precondition against ischemia reperfusion injury in mice. Aging Cell.

[115] Van Ginhoven T.M., Mitchell J.R., Verweij M., Hoeijmakers J.H., Ijzermans J.N., de Bruin R.W. (2009). The use of preoperative nutritional interventions to protect against hepatic ischemia-reperfusion injury. Liver Transp..

[116] Varendi K., Airavaara M., Anttila J., Vose S., Planken A., Saarma M., Mitchell J.R., Andressoo J.O. (2014). Short-term preoperative dietary restriction is neuroprotective in a rat focal stroke model. PLoS ONE.

[117] Lee J., Duan W., Mattson M.P. (2002). Evidence that brain-derived neurotrophic factor is required for basal neurogenesis and mediates, in part, the enhancement of neurogenesis by dietary restriction in the hippocampus of adult mice. J. Neurochem..

[118] Zhong F., Jiang Y. (2019). Endogenous Pancreatic β Cell Regeneration: A Potential Strategy for the Recovery of β Cell Deficiency in Diabetes. Front. Endocrinol. (Lausanne).

[119] Yu Z.F., Mattson M.P. (1999). Dietary restriction and 2-deoxyglucose administration reduce focal ischemic brain damage and improve behavioral outcome: Evidence for a preconditioning mechanism. J. Neurosci. Res..

[120] Lee C., Safdie F.M., Raffaghello L., Wei M., Madia F., Parrella E., Hwang D., Cohen P., Bianchi G., Longo V.D. (2010). Reduced levels of IGF-I mediate differential protection of normal and cancer cells in response to fasting and improve chemotherapeutic index. Cancer Res..

[121] Berryman D.E., Christiansen J.S., Johannsson G., Thorner M.O., Kopchick J.J. (2008). Role of the GH/IGF-1 axis in lifespan and healthspan: Lessons from animal models. Growth Horm. IGF Res..

[122] Fabrizio P., Pozza F., Pletcher S.D., Gendron C.M., Longo V.D. (2001). Regulation of longevity and stress resistance by Sch9 in yeast. Science.

[123] Longo V.D. (1999). Mutations in signal transduction proteins increase stress resistance and longevity in yeast, nematodes, fruit flies, and mammalian neuronal cells. Neurobiol. Aging.

[124] Raffaghello L., Lee C., Safdie F.M., Wei M., Madia F., Parrella E., Hwang D., Cohen P., Bianchi G., Longo V.D. (2008). Starvation-dependent differential stress resistance protects normal but not cancer cells against high-dose chemotherapy. Proc. Natl. Acad. Sci. USA.

[125] Swindell W.R. (2007). Gene expression profiling of long-lived dwarf mice: Longevity-associated genes and relationships with diet, gender and aging. BMC Genomics.

[126] Hanahan D., Weinberg R.A. (2011). Hallmarks of cancer: The next generation. Cell.

[127] Mitra M.S., Donthamsetty S., White B., Latendresse J.R., Mehendale H.M. (2007). Mechanism of protection of moderately diet restricted rats against doxorubicin-induced acute cardiotoxicity. Toxicol. Appl. Pharmacolol..

[128] Di Biase S., Shim H.S., Kim K.H., Vinciguerra M., Rappa F., Rappa F., Wei M., Brandhorst S., Cappello F., Mirzaei H. (2017). Fasting regulates EGR1 and protects from glucose- and dexamethasone-dependent sensitization to chemotherapy. PLoS Biol..

[129] Cheng C.W., Adams G.B., Perin L., Wei M., Zhou X., Lam B.S., Da Sacco S., Mirisola M., Quinn D.I., Dorff T.B. (2014). Prolonged fasting reduces IGF-1/PKA to promote hematopoietic stem cell-based regeneration and reverse immunosuppression. Cell Stem Cell.

[130] Shi Y., Felley-Bosco E., Marti T.M., Orlowski K., Pruschy M., Stahel R.A. (2012). Starvation-induced activation of ATM/Chk2/p53 signaling sensitizes cancer cells to cisplatin. BMC Cancer.

[131] Blagosklonny M.V., Pardee A.B. (2001). Exploiting cancer cell cycling for selective protection of normal cells. Cancer Res..

[132] Jongbloed F., Huisman S.A., van Steeg H., Pennings J.L.A., IJzermans J.N.M., Dollé M., de Bruin R. (2019). The transcriptomic response to irinotecan in colon carcinoma bearing mice preconditioned by fasting. Oncotarget.

[133] Huisman S.A., Bijman-Lagcher W., IJzermans J.N., Smits R., de Bruin R.W. (2015). Fasting protects against the side effects of irinotecan but preserves its anti-tumor effect in Apc15lox mutant mice. Cell Cycle.

[134] Kawaguchi T., Takemura G., Kanamori H., Takeyama T., Watanabe T., Morishita K., Ogino A., Tsujimoto A., Goto K., Maruyama R. (2012). Prior starvation mitigates acute doxorubicin cardiotoxicity through restoration of autophagy in affected cardiomyocytes. Cardiovasc. Res..

[135] Brandhorst S., Wei M., Hwang S., Morgan T.E., Longo V.D. (2013). Short-term calorie and protein restriction provide partial protection from chemotoxicity but do not delay glioma progression. Exp. Gerontol..

[136] Tinkum K.L., Stemler K.M., White L.S., Loza A.J., Jeter-Jones S., Michalski B.M., Kuzmicki C., Pless R., Stappenbeck T.S., Piwnica-Worms D. (2015). Fasting protects mice from lethal DNA damage by promoting small intestinal epithelial stem cell survival. Proc. Natl. Acad. Sci. USA.

[137] Caffa I., Spagnolo V., Vernieri C., Valdemarin F., Becherini P., Wei M., Brandhorst S., Zucal C., Driehuis E., Ferrando L. (2020). Fasting-mimicking diet and hormone therapy induce breast cancer regression. Nature.

[138] Rebucci M., Michiels C. (2013). Molecular aspects of cancer cell resistance to chemotherapy. Biochem. Pharmacol..

[139] Luqmani Y.A. (2005). Mechanisms of drug resistance in cancer chemotherapy. Med. Princ. Pract..

[140] Gurunathan S., Kang M.H., Qasim M., Kim J.H. (2018). Nanoparticle-Mediated Combination Therapy: Two-in-One Approach for Cancer. Int. J. Mol. Sci..

[141] Simone B.A., Palagani A., Strickland K., Ko K., Jin L., Lim M.K., Dan T.D., Sarich M., Monti D.A., Cristofanilli M. (2018). Caloric restriction counteracts chemotherapy-induced inflammation and increases response to therapy in a triple negative breast cancer model. Cell Cycle.

[142] Safdie F., Brandhorst S., Wei M., Safdie F., Brandhorst S., Hwang S., Conti P.S., Chen T.C., Longo V.D. (2012). Fasting enhances the response of glioma to chemo- and radiotherapy. PLoS ONE.

[143] Bianchi G., Martella R., Ravera S., Marini C., Capitanio S., Orengo A., Emionite L., Lavarello C., Amaro A., Petretto A. (2015). Fasting induces anti-Warburg effect that increases respiration but reduces ATP-synthesis to promote apoptosis in colon cancer models. Oncotarget.

[144] Pietrocola F., Pol J., Vacchelli E., Rao S., Enot D.P., Baracco E.E., Levesque S., Castoldi F., Jacquelot N., Yamazaki T. (2016). Caloric Restriction Mimetics Enhance Anticancer Immunosurveillance. Cancer Cell.

[145] Pietrocola F., Pol J., Vacchelli E., Baracco E.E., Levesque S., Castoldi F., Maiuri M.C., Madeo F., Kroemer G. (2016). Autophagy induction for the treatment of cancer. Autophagy.

[146] Di Biase S., Lee C., Brandhorst S., Manes B., Buono R., Cheng C.W., Cacciottolo M., Martin-Montalvo A., de Cabo R., Wei M. (2016). Fasting-Mimicking Diet Reduces HO-1 to Promote T Cell-Mediated Tumor Cytotoxicity. Cancer Cell.

[147] Sun P., Wang H., He Z., Chen X., Wu Q., Chen W., Sun Z., Weng M., Zhu M., Ma D. (2017). Fasting inhibits colorectal cancer growth by reducing M2 polarization of tumor-associated macrophages. Oncotarget.

[148] D’Aronzo M., Vinciguerra M., Mazza T., Panebianco C., Saracino C., Pereira S.P., Graziano P., Pazienza V. (2015). Fasting cycles potentiate the efficacy of gemcitabine treatment in in vitro and in vivo pancreatic cancer models. Oncotarget.

[149] Shim H.S., Wei M., Brandhorst S., Longo V.D. (2015). Starvation promotes REV1 SUMOylation and p53-dependent sensitization of melanoma and breast cancer cells. Cancer Res..

[150] Lu Z., Xie J., Wu G., Shen J., Collins R., Chen W., Kang X., Luo M., Zou Y., Huang L.J. (2017). Fasting selectively blocks development of acute lymphoblastic leukemia via leptin-receptor upregulation. Nat. Med..

[151] Siggens L., Figg N., Bennett M., Foo R. (2012). Nutrient deprivation regulates DNA damage repair in cardiomyocytes via loss of the base-excision repair enzyme OGG1. FASEB J..

[152] Witkamp R.F., van Norren K. (2018). Let thy food be thy medicin when possible. Eur. J. Pharmacol..

[153] Vernieri C., Casola S., Foiani M., Pietrantonio F., de Braud F., Longo V. (2016). Targeting Cancer Metabolism: Dietary and Pharmacologic Interventions. Cancer Discov..

[154] Tannenbaum A. (1947). Effects of varying caloric intake upon tumor incidence and tumor growth. Ann. N. Y. Acad. Sci..

[155] Weindruch R., Walford R.L. (1982). Dietary restriction in mice beginning at one year of age. Effects on life span and spontaneous cancer incidence. Science.

[156] Levine M.E., Suarez J.A., Brandhorst S., Balasubramanian P., Cheng C.W., Madia F., Fontana L., Mirisola M.G., Guevara-Aguirre J., Wan J. (2014). Low protein intake is associated with a major reduction in IGF-1, cancer, and overall mortality in the 65 and younger but not older population. Cell Metab..

[157] Erickson N., Boscheri A., Linke B., Huebner J. (2017). Systematic review: Isocaloric ketogenic dietary regimes for cancer patients. Med. Oncol..

[158] Turbitt W.J., Demark-Wahnefried W., Peterson C.M., Norian L.A. (2019). Targeting Glucose Metabolism to Enhance Immunotherapy: Emerging Evidence on Intermittent Fasting and Calorie Restriction Mimetics. Front. Immunol..

[159] Bader J.E., Voss K., Rathmell J.C. (2020). Targeting Metabolism to Improve the Tumor Microenvironment for Cancer Immunotherapy. Mol. Cell..

[160] Fontana L., Adelaiye R.M., Rastelli A.L., Miles K.M., Ciamporcero E., Longo V.D., Nguyen H., Vessella R., Pilli R. (2013). Dietary protein restriction inhibits tumor growth in human xenograft models. Oncotarget.

[161] Yu D., Yang S.E., Miller B.R., Wisinski J.A., Sherman D.S., Brinkman J.A., Tomasiewicz J.L., Cummings N.E., Kimple M.E., Cryns V.L. (2018). Lamming Short-term methionine deprivation improves metabolic health via sexually dimorphic, mTORC1-independent mechanisms. FASEB J..

[162] Klement R.J., Champ C.E., Otto C., Kämmerer U. (2016). Anti-Tumor Effects of Ketogenic Diets in Mice: A Meta-Analysis. PLoS ONE.

[163] Haas J.T., Staels B. (2017). Fasting the Microbiota to Improve Metabolism?. Cell Metab..

[164] Caffa I., D’Agostino V., Damonte P., Soncini D., Cea M., Monacelli F., Odetti P., Ballestrero A., Provenzani A., Longo V.D. (2015). Fasting potentiates the anticancer activity of tyrosine kinase inhibitors by strengthening MAPK signaling inhibition. Oncotarget.

[165] Lo Re O., Panebianco C., Porto S., Cervi C., Rappa F., Di Biase S., Michele Caraglia M., Pazienza V., Manlio Vinciguerra M. (2018). Fasting inhibits hepatic stellate cells activation and potentiates anti-cancer activity of Sorafenib in hepatocellular cancer cells. Cell Physiol..

[166] Bragazzi N.L., Briki W., Khabbache H., Rammouz I., Chamari K., Demaj T., Re T.S., Zouhir M. (2016). Ramadan Fasting and Patients with Cancer: State-of-the-Art and Future Prospects. Front. Oncol..

[167] Adawi M., Watad A., Brown S., Aazza K., Aazza H., Zouhir M., Sharif K., Ghanayem K., Farah R., Mahagna H. (2017). Ramadan Fasting Exerts Immunomodulatory Effects: Insights from a Systematic Review. Front. Immunol..

[168] Bragazzi N.L., Sellami M., Salem I., Conic R., Kimak M., Pigatto P.D.M., Damiani G. (2019). Fasting and Its Impact on Skin Anatomy, Physiology, and Physiopathology: A Comprehensive Review of the Literature. Nutrients.

[169] Damiani G., Mahroum N., Pigatto P.D.M., Pacifico A., Malagoli P., Tiodorovic D., Conic R.R., Amital H., Bragazzi N.L., Watad A. (2019). The Safety and Impact of a Model of Intermittent, Time-Restricted Circadian Fasting (“Ramadan Fasting”) on Hidradenitis Suppurativa: Insights from a Multicenter, Observational, Cross-Over, Pilot, Exploratory Study. Nutrients.

[170] Active Clinical Trials Addressing Fasting in Oncology. https://clinicaltrials.gov.

[171] Lévesque S., Pol J.G., Ferrere G., Galluzzi L., Zitvogel L., Kroemer G. (2019). Trial watch: Dietary interventions for cancer therapy. Oncoimmunology.

